# Cracking the Code: Computational Image Analysis Tools for Histopathological and Morphometric Insights

**DOI:** 10.3390/jimaging12040173

**Published:** 2026-04-17

**Authors:** Ana Luisa Teixeira de Almeida, Ana Beatriz Gram dos Santos, Debora Ferreira Barreto-Vieira

**Affiliations:** Laboratory of Viral Morphology and Morphogenesis, Oswaldo Cruz Institute, Oswaldo Cruz Foundation Fiocruz, Rio de Janeiro 21040-900, RJ, Brazil; gramanabeatriz@gmail.com

**Keywords:** computational histopathology, morphometry, image analysis software

## Abstract

The assessment of histopathological features has evolved considerably, transitioning from traditional manual measurements to more sophisticated, technology-assisted approaches. Classical histological evaluation, while foundational and highly reliable, is inherently labor-intensive and subject to inter-observer variability. With the advent of digital pathology, these practices have been progressively enhanced by image processing software, which offers capabilities such as segmentation, feature extraction, and data visualization. However, despite their promise, the integration of machine learning into this branch of pathology faces notable challenges, such as the need for large, high-quality annotated datasets and the integration into existing workflows, which remain significant hurdles. Looking forward, the role of specialists in histological evaluation remains crucial in this evolving landscape. While automation streamlines routine tasks, the expertise of pathologists is indispensable in validating results and interpreting findings in scientific contexts. This comprehensive review explores the trajectory of histological evaluation methods, from manual and classical strategies to cutting-edge digital tools, highlighting the benefits, limitations, and implications of each approach in contemporary practice.

## 1. Introduction

In the field of morphometry, which involves quantitative analysis of the shape and size of structures, different variables are utilized to characterize features and dimensions [1]. These variables include linear measurements, areas, perimeters, ratios, and geometric descriptors, all of which can contribute to a more objective understanding of tissue architecture. For several decades, morphologists and pathologists have relied on quantitative morphometric techniques as essential tools in histological and pathological assessments, providing measurable data to support visual observations [2,3].

More recently, advances in digital pathology and image analysis have propelled a growing trend toward the integration of these quantitative approaches in the investigation of tissue composition and disease processes. These methods are now widely used to evaluate how pathological conditions, environmental exposures, and physiological changes influence the structure and function of tissues, ultimately contributing to diagnostic precision and research reproducibility [4,5]. Through these analyses, researchers can obtain valuable insights into the normal organization of biological systems and detect subtle alterations induced by internal or external stimuli, which may not be readily apparent through qualitative assessment alone.

In the context of histological slides, morphometric evaluation can be applied to a variety of parameters, including the measurement of linear dimensions (such as thickness or length of tissue layers), surface areas of cells or compartments, and proportional relationships between different tissue elements. These quantifications play a critical role in comparative studies, disease classification, and the identification of histological biomarkers [6,7]. As such, morphometry has become a cornerstone in both fundamental research and applied biomedical sciences, enhancing our capacity to interpret tissue morphology in a systematic and reproducible manner.

## 2. Do Numbers Lie?

“Numbers don’t lie”. This well-known saying emphasizes the reliability of quantitative data, particularly in fields that have traditionally relied on qualitative assessments, such as pathology. In the realm of histopathological examination, the integration of quantitative parameters, or at least the semi-quantitative analysis, offers much-needed objectivity [8]. While subjective interpretations of cellular and tissue structures can lead to inconsistencies, employing metrics and statistical methods enhances accuracy [9]. The conversion of qualitative observations into quantifiable measurements improves the reliability of findings and facilitates comparisons that are more robust across studies [10].

However, morphometrics encounters several challenges that have always been critical to achieving robust and meaningful results [11]. Among these, the number of analyzed fields or individuals plays a crucial role, as it directly affects both the robustness and the generalizability of the findings. Intraspecific variation, where individuals within the same species vary in size and shape, also poses challenges in capturing overall patterns and necessitates careful sampling strategies to encompass morphological diversity effectively [12]. Furthermore, sampling errors, often arising from non-representative sampling methods, can compromise the validity of conclusions drawn about the morphology of the population under study [13].

Standardizing measurement methods is essential to ensure valid comparisons across different studies, highlighting the importance of precise measurements that may vary depending on equipment and operator skill [14]. Adequate sample size is crucial, as a small sample may not be statistically representative, thereby failing to capture morphological variability within the population [15].

## 3. Complexity in Life

Morphometric analyses of inanimate materials often benefit from greater structural regularity. These materials typically possess more uniform and homogeneous shapes, allowing for simpler analyses and the application of traditional statistical methods. When quantifying the morphometric properties of crystalline structures, for instance, researchers can rely on well-defined geometric parameters, such as grain size, aspect ratio, and surface area, which may exhibit less variability compared to biological specimens [16].

The imaging techniques for inanimate materials can capture morphology more directly and accurately, using methods like tomography or microtomography. These techniques provide high-resolution 3D images that allow for detailed analyses of features, such as porosity, density, and surface roughness [17]. Such quantifications are crucial in material sciences for applications like assessing the performance of composites, metals, and polymers, where precise morphometric data can inform mechanical properties and suitability for specific applications [18].

In contrast, biological specimens exhibit significant natural variation both within and across individuals. The dynamic nature of cells, including their movement and shape changes, adds further complexity to capturing accurate morphometric data at specific time points [19,20]. Moreover, imaging techniques such as optical, confocal, and electron microscopies and tomography have limitations in terms of depth of penetration, resolution, contrast, and the ability to faithfully represent three-dimensional structures [21,22].

Morphometric evaluations of cellular and tissue sections encounter specific challenges. The first challenge is the limited spatial resolution depending on the imaging technique used, hindering detailed analysis of very small cellular structures or subcellular details [23]. Interpreting the dimensions of cells and tissue structures presents another complexity due to their three-dimensional nature and the difficulty in accurately capturing their exact shape in a single two-dimensional projection, usually resorting to prediction models [24].

Image acquisition systems aim to achieve the best representation of a real object. However, the acquisition process can lead to losses in final quality due to compression artifacts and physical limitations, for which algorithms can be employed to perform image deconvolution and minimize artifacts, including noise and blur [25]. Consequently, advancements in high-resolution imaging have led to the generation of large datasets, necessitating the development of effective methods for handling and analyzing extensive morphometric data [26].

**Figure 1:**
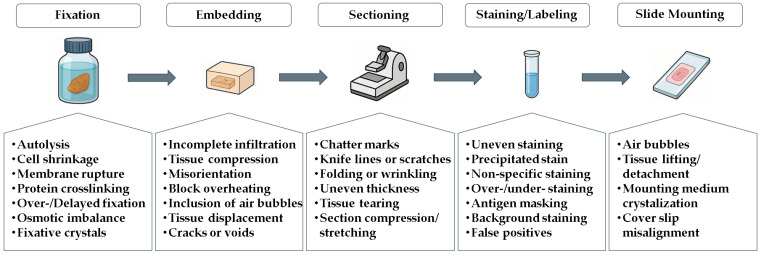
Source of artifacts in histological sample preparation.

Additionally, artifacts can be introduced during the sample preparation process (Figure 1), affecting the structural features of cell cultures or tissues at various stages, including fixation, embedding, sectioning, staining or labeling, and slide mounting [27]. Histological samples can vary significantly in quality and preparation, which poses challenges for accurate analysis. However, pathologists and morphologists are trained to handle these variations and adjust their analyses accordingly, ensuring reliable results. When faced with unclear images or ambiguous interpretations, they may employ additional techniques, such as special staining or immunohistochemistry, to enhance the clarity and precision of their assessments [28].

## 4. Morphometry: The Old, the New, and the Borrowed

Manual morphometry, which involves the measurement and analysis of anatomical features through manual methods, faces various limitations and challenges. One significant issue is observer bias, where data interpretation can differ among observers. Even intra-observer variability can occur; therefore, to ensure the reliability of collected data, it is essential to implement a variety of tests. An intra-observer comparison can be performed using the intraclass correlation coefficient (ICC) to evaluate measurement deviations across distinct assessments for each variable. This quantifies the agreement among repeated observations by the same observer and identifies variability arising from subjective interpretation or measurement techniques [29].

To further enhance the reliability of findings, complementary methodologies can be utilized. Repeated measures ANOVA is particularly effective for assessing the agreement between two different measurement methods or among various observers [30]. By plotting the differences against the means of these measurements, using Bland–Altman analysis, for instance, we can visually illustrate the level of agreement and help identify any systematic biases [31].

These approaches provide additional insights into the consistency and accuracy of measurements, allowing researchers to detect potential biases and refine their measurement protocols. Additionally, conducting training sessions for observers can significantly reduce intra-observer variability, thereby improving the overall validity of the study [32]. This validation is especially relevant because any subjectivity is particularly problematic in biological studies, where slight variations in interpretation can significantly impact conclusions or even diagnoses.

Moreover, the manual method is labor-intensive and time-consuming, particularly when dealing with extensive datasets or complex features that require numerous measurements. Researchers often need to manually measure hundreds or even thousands of specimens, a process that not only depends heavily on the observer’s skill and experience but also increases the risk of fatigue-related errors [33]. Such reliance on human expertise often results in less accuracy compared to automated or semi-automated methods, which can utilize advanced algorithms to standardize measurements and reduce variability and increase scalability [34].

Even so, the expertise of researchers remains invaluable in shaping the analysis and accurately interpreting complex biological features, such as three-dimensional data. Meanwhile, the adoption of automated and semi-automated morphometric methods significantly enhances the efficiency and precision of data collection [35]. This allows scientists to focus more on interpreting results rather than spending excessive time on data collection.

When comparing manual quantification performed by experienced professionals with the use of algorithms and ML, it is essential to weigh the advantages and disadvantages of each approach. Manual quantification leverages the expertise of professionals, who can identify subtle details and ensure accurate measurements, particularly in unconventional or complex scenarios [36]. This method also offers flexibility, allowing for real-time adjustments to capture specific data, and the visual validation of measurements enhances the understanding of morphological characteristics.

On the other hand, some software platforms may have a steep learning curve and require technical proficiency for effective operation and optimization. However, it is essential to highlight that observer bias is an inherent challenge that cannot be fully eliminated through any approach. This bias may persist even in methods that do not directly engage an observer, such as automated assessments based on image analysis, which merely redistribute the bias to different sources. In these cases, the potential for observer bias is merely shifted to the individual who previously calibrated the automated system or who trained the algorithm [37].

## 5. Fundamentals of Image Analysis Software in Morphometry

Morphometric analysis employs a combination of image processing techniques and algorithms to extract quantitative measurements of morphological features from digital images. The process begins with importing digital images intended for analysis, which can be obtained from optical and electron microscopies or from other digital imaging techniques, such as computed tomography and magnetic resonance imaging. Once the images are imported, they may undergo preprocessing steps such as color correction, contrast adjustment, and noise reduction [38]. These steps enhance image quality and facilitate subsequent segmentation.

In order to extract information from specific structures, it is essential to segment them from the entirety of the image. Segmentation is a crucial phase in the analysis, where software identifies and delineates areas of interest, such as defined areas, whole cells, organelles, and microorganisms. This process can be carried out automatically, semi-automatically, or manually, depending on the image complexity and the software utilized (Table 1) [39]. After segmentation, the software can extract various morphological features from the identified regions, including area, perimeter, shape, and color intensity.

These image processing programs offer a range of additional capabilities that enhance user experience and analytical power, such as plugins and macros, which serve distinct yet complementary roles in boosting functionality. Plugins are additional extensions that enrich the software by integrating specialized features, such as advanced segmentation algorithms or sophisticated pattern recognition tools [49,50]. In contrast, macros are predefined sequences of commands designed to automate repetitive or complex tasks within the software. This automation capability facilitates streamlined workflows, allowing users to record a series of actions and replay them as needed [51]. Consequently, macros enable consistent and rapid analysis of extensive image datasets. For example, they can automate processes such as image loading, filter application, object segmentation, feature measurement, and result exportation.

Scripting, on the other hand, involves the creation and execution of small programs that automate specific tasks within the software environment. Typically written in programming languages supported by the software, scripts enable users to manipulate data and perform advanced image analysis operations [52].

Additionally, workflows, defined as organized sequences of steps or operations, allow users to automate complex image analysis processes. For instance, workflows can be designed to perform multifaceted analyses that include segmentation, texture analysis, and pattern classification in a single execution. By combining user-friendly tools and customizable pipelines, these workflows can be adapted to various imaging technologies and research questions, supporting applications ranging from spatial profiling of cell phenotypes to the detection of cellular neighborhoods and cell–cell interactions [53]. Overall, the integration of macros, scripts, and workflows in image processing software significantly enhances the efficiency of analysis and empowers researchers to explore their data more deeply.

To instruct the software on what to measure, a structured approach is followed. First, specific parameters for segmentation and analysis are configured, such as intensity thresholds or object size criteria. Next, regions of interest (ROIs) within the image are defined, either manually or automatically, specifying where the software should focus its analysis efforts. In advanced cases, some software platforms support the training of algorithms, enabling the software to automatically recognize specific features based on provided examples [54].

After the initial analysis is completed, it is essential to validate the results by comparing them with manual measurements or established knowledge [55,56]. If necessary, adjustments to the analysis parameters are made to improve accuracy. Depending on the complexity of the analysis or the characteristics of the images, multiple iterations of segmentation may be required to achieve satisfactory results [57].

Once the data are collected, the final stages of the process are crucial for effective communication and interpretation of the findings. These results are typically presented through a variety of formats, including graphs, tables, and visualizations, which serve to elucidate underlying patterns and relationships within the data. For instance, scatter plots can illustrate correlations between different morphometric parameters [58], while histograms can provide insights into the distribution of measurements across the sample population [59]. Heatmaps serve as a valuable tool to enhance the visualization of concentration patterns or variations in morphometric traits, making complex data more accessible across diverse fields such as radiological imaging, high-resolution phenotyping, and artificial intelligence (AI) explainability [60,61,62].

## 6. Morphometric Analysis of Histopathological Findings

In the field of histopathological analysis, most studies make use of whole-slide imaging (WSI), a cutting-edge technology that has revolutionized the field of pathology by enabling the digitalization of entire histological slides [63]. The process of obtaining whole-slide images involves several meticulous steps, and specialized equipment and software that collectively facilitate the analysis and interpretation of pathological specimens.

Once the tissue slides are prepared, the next step involves the use of a whole-slide scanner, an advanced imaging device that captures high-resolution digital images of the slides. These scanners operate via a process known as image tiling, in which the scanner systematically captures multiple overlapping images of the slide at various focal depths. This process is essential for creating a seamless, high-resolution image that represents the entire slide. Modern WSI scanners utilize various technologies, including brightfield and fluorescence imaging, to accommodate different staining methods and to capture specific cellular markers [64].

The resulting images are then processed using specialized software that stitches the individual images together, creating a comprehensive and cohesive digital representation of the histological slide [64]. Subsequently, the resulting images are processed using pre-processing techniques, including grayscale and color normalization, resampling, denoising, and contrast enhancement, that are commonly applied to harmonize images from different sources or devices [65]. These adjustments standardize image appearance, intensity, and resolution to enable consistent analyses but require careful implementation and rigorous quality control to prevent artifacts or inconsistencies that could compromise result reliability.

This type of software often incorporates advanced features such as image analysis tools, enabling pathologists to perform quantitative assessments, annotate regions of interest, and implement algorithms for diagnostic purposes. Recent developments in digital pathology include flexible, open-source platforms that support a wide range of deep learning methods, offering functionalities like stain normalization, data augmentation, whole-slide classification, uncertainty quantification, feature extraction, and real-time visualization [66]. These tools are optimized for efficient processing of high-resolution images and often feature intuitive graphical interfaces that facilitate experimentation and deployment across diverse hardware environments.

Despite the numerous advantages of WSI, not all histological studies utilize this methodology. Several factors contribute to this decision. Firstly, the cost of whole-slide scanners and the associated software can be prohibitively high, particularly for smaller laboratories or institutions with limited funding [64]. In certain cases, pathologists may choose to focus on specific areas of interest, utilizing traditional microscopy techniques to examine targeted regions of the slide. This approach may be sufficient for certain diagnostic purposes, particularly when known lesions or abnormalities are present.

One of the significant benefits of employing WSI is the considerable reduction in time analysis compared to random field images in morphometric studies [67]; however, by strategically selecting specific areas of interest, researchers can expedite the diagnostic process, which is particularly advantageous when dealing with large sample sizes, thus resulting in a slightly faster analysis [68].

Histopathological specimens often exhibit heterogeneity, with varying degrees of pathological features dispersed throughout different regions of the tissue, and the challenge lies in ensuring that sampling from random fields is sufficiently representative [69]. Consequently, relying solely on randomly selected fields may fail to adequately encapsulate the complete spectrum of morphological changes, leading to potentially skewed interpretations of histological characteristics. This concern is especially relevant in the context of diseases marked by focal lesions or patchy distributions, where critical areas of pathology may be overlooked [70].

Furthermore, considerations regarding sample size and statistical power become paramount in this context. While random sampling can enhance operational efficiency, it simultaneously raises questions about whether the sample size is sufficient for drawing robust conclusions. In the diagnostic scope, a focused analysis can reduce the applied time; however, a quantitative analysis that relies on small sample sizes may introduce undesirable sampling errors in morphometric studies. Conducting power analyses is essential to confirm that the selected fields provide an adequate dataset for reliable statistical evaluations. Additionally, the inherent variability among the sampled fields necessitates meticulous attention to the sampling strategy employed to circumvent the risks of committing type I and type II errors during hypothesis testing [71].

As previously mentioned, not all studies have the infrastructure or work model based on WSI analysis. However, an important point that needs to be considered is sampling. Certain information should be provided to ensure reproducibility and clarity in the acquisition of data. The area of each field or the total area evaluated is a commonly dismissed topic; however, the sizes of these fields can vary significantly depending on the image capture system employed.

For instance, Zravy and colleagues conducted a detailed analysis to characterize the inflammatory response in the brains of septic patients, highlighting the significant impact of systemic inflammation on human brain tissue. The authors detailed their methodology for morphometric assessment, which involved capturing images from 30 randomly selected fields, each measuring 0.9126 mm^2^, at a 10× objective lens magnification (0.43 mm^2^) and processing them using ImageJ software [72], relevant data that should be reported in studies employing this type of methodology.

This raises another important issue: How many fields are deemed reasonable to allow for reliable generalization of the observed results? Only a limited number of sections from a given organ are typically examined, meaning that the analysis cannot encompass the entire organ. Therefore, the total number of fields analyzed becomes critical to ensure that, at least within the evaluated section, the full range of potential morphological alterations is adequately accounted for. In the literature, a total of 30 fields is frequently cited as a reference in morphometric studies involving random field selection [73,74,75]. This number is not arbitrary; it aligns with statistical principles, particularly concerning the Central Limit Theorem, which suggests that sample sizes of 30 or more tend to produce distributions that approximate normality [76,77].

While studies with smaller sample sizes, such as those with 10 fields [78,79] or even 5 fields [80], are not uncommon, they do raise the risk of making inaccurate generalizations. Keeping this in mind, we will present a case study aimed at discussing random field selection in histopathological analysis and the level of variation obtained from these assessments.

## 7. Sampling Effects on Morphometric Analysis: An Illustrative Example

Morphometric analyses are widely used to quantify tissue alterations in experimental models and clinical samples. However, the reliability of these measurements strongly depends on how histological fields are sampled, in terms of both number and spatial arrangement. Small or narrowly localized samples often fail to capture the heterogeneity of tissue pathology, which increases the risk of biased conclusions.

The liver provides a particularly illustrative example of these challenges. It is a major target organ in dengue virus infection [81] and, more generally, in many systemic diseases. Histological alterations in this tissue are frequently multifocal and variable in distribution. One of the most prominent morphological alterations observed during liver infection is hepatocyte death [82]. The use of automated tools for quantifying these cells poses challenges, especially in cases involving coagulative necrosis. In such instances, although nuclear structures may remain visible, the cells are no longer viable. Thus, tools that rely solely on nuclear recognition for cell counting are inadequate for distinguishing functional cells or quantifying parameters such as the proportion of necrotic tissue in each field.

Another variable examined was the proportion of binucleated hepatocytes. Binucleation is a known cellular response to tissue injury and viral infection, reflecting an increased demand for protein synthesis [83,84]. However, in practical scenarios, even in well-preserved samples, cell boundaries may be indistinct. As a result, automatic recognition tools based on deep learning, even when appropriately trained, may exhibit a significant error rate, highlighting the essential role of the morphologist in evaluating tissue changes in certain contexts.

The proportion of the area occupied by sinusoidal capillaries was quantified as well. Infection-related responses may lead to either the collapse or expansion of these vascular spaces, ultimately resulting in disorganization of the typical hepatic cord architecture [85]. This variable was assessed using the thresholding tool in ImageJ software for area segmentation, followed by calculation of the relative proportion of the segmented area to the total image field.

Different image subsets were systematically selected to evaluate variability in outcomes related to total hepatocyte counts, percentage of bi-/multi-nucleated hepatocytes, and the proportion of sinusoidal capillary area per field. The subsets included 10, 30, or 40 images from the same sectioning plane; 30 images comprising 10 from each of the three non-successive sectioning planes; and the full dataset of 120 images spanning all three planes. Methodological details, statistical comparisons, and full graphical outputs supporting this illustrative case are provided in the Supplementary Materials.

All quantifications were performed manually, as introducing automated analysis methods would have added an additional layer of variability, potentially stemming from factors such as inadequate training datasets, limitations in algorithm generalization, and recognition errors related to subtle morphological features. All measurements were carried out by a single trained observer, and blinding procedures were not adopted. Although we acknowledge the value of blinding and observer reliability assessments, these approaches were beyond the scope of our objectives. It is important to emphasize that these within-animal measurements serve not for direct population inference but as a methodological assessment of within-slide sampling strategies, recognizing they are not statistically independent observations.

**Figure 2:**
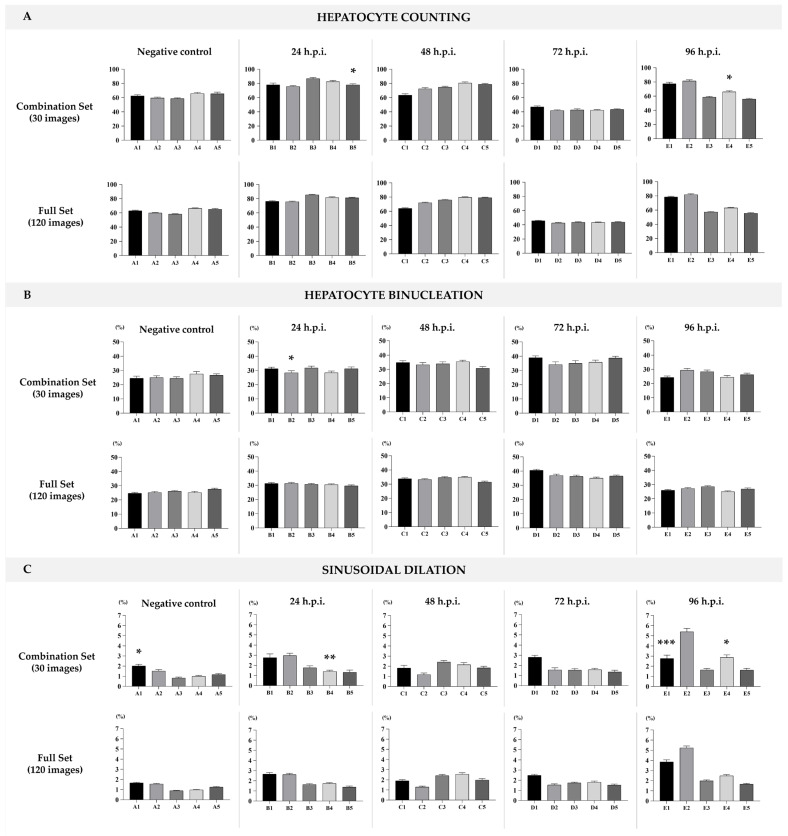
(**A**) Total hepatocytes count per field. (**B**) Percentage of bi-/multi-nucleated hepatocytes per field. (**C**) Proportion of luminal space in sinusoidal capillaries. Top row: graphical representation based on a combination of 30 images per animal (10 images from each of the three sectioning planes), with one graph for each experimental time point: negative control, 24 h, 48 h, 72 h, and 96 h post-infection (h.p.i.). Statistical analysis: unpaired *t*-test with Welch’s correction. * *p* < 0.05; ** *p* < 0.01; *** *p* < 0.001. Bottom row: corresponding graphs based on the full dataset of 120 images per group, representing the total sampling for each time point.

In particular, sampling 30 images evenly across multiple sectioning planes approximated the full dataset with high fidelity, while requiring substantially less analytical effort (Figure 2A–C). In comparison, analyses based on very few fields (e.g., 5 or 10) tended to underestimate biological variability and could lead to false-negative conclusions. In our case, when comparing the 10-image sampling against the remaining dataset, some tests did not reach statistical significance (see Supplementary Material Data File S1). However, this lack of significance should not be interpreted as equivalence; rather, it likely reflects the limited statistical power and representativeness of smaller samples.

This example illustrates two central considerations for morphometric analysis. The first is that the representativeness of tissue sampling is as critical as the measurement technique itself. The second is that applying the same field selection strategy across different organs or lesion models requires caution, as variations in tissue architecture and injury patterns may necessitate adjustments to ensure methodological consistency and comparability. The interplay between sample size, statistical power, and sectioning planes will be critical in refining our analysis and ensuring that our findings are both statistically valid and clinically relevant.

## 8. Semi-Quantitative Analysis

In the field of histopathology, grading tissue alterations is crucial for diagnosing and prognosticating various diseases, particularly cancers. Semi-quantitative assessment strikes a balance between qualitative descriptions and quantitative measurements [86]. This method is especially valuable in animal models for diseases, where straightforward numerical data may not adequately capture the nuances of tissue changes, highlighting the need for improved translation of these qualitative changes to more effectively correlate them with human disease [28].

Semi-quantitative scoring systems typically combine qualitative descriptors with numerical grades or ranges to categorize the severity of histopathological alterations [87]. For instance, such a system might assess the severity of a lesion on a scale from 0 to 3, where a score of 0 indicates the absence of change, a 1 denotes a “mild” change, a 2 represents a “moderate” change, and a 3 signifies a “severe” alteration [88]. A similar pattern can be employed to characterize the extent or distribution of alterations, with values ranging from 0 to 3, indicating absence, focal presence, multifocal presence, and diffuse presence, respectively [86]. This component tends to be more subjective.

Alternatively, in the context of grading immunohistochemistry staining or lesion extent, a ranking based on proportion may be utilized. For example, numerical assignments can be made as 0 for no staining, 1 for less than 10% of cells staining, 2 for staining in 10% to 50%, and 3 for greater than 50% of cells staining [89]. The same reasoning can be applied to the extent of morphological changes, where the percentage of the image in which the alteration is detected is measured and subsequently assigned to a score grade.

However, semi-quantitative methods have notable disadvantages. The subjectivity in assigning scores may introduce variability among observers, affecting reliability. Additionally, these methods may oversimplify histological features, potentially leading to a loss of critical information [90]. To enhance reliability, it is crucial to refine scoring systems. Therefore, studies employing semi-quantitative grading should clearly describe the specific criteria used for each grade, detailing the histological features evaluated, grading thresholds, and any supplementary techniques employed, such as immunohistochemical staining or molecular profiling [91].

## 9. AI-Powered Analysis: Machine Learning Key Concepts

The implementation of machine learning (ML) and the design of pipelines within morphometry software is a complex process that relies heavily on the specific capabilities of each platform. As previously discussed, morphometric analyses typically involve handling substantial amounts of data, especially when WSI is used, whose large file sizes can pose significant challenges for long-term storage and local processing or analysis [92]. A study published in 2020 highlighted the application of morphometry on lung sections from COVID-19 patients, where over 9000 fields were assessed for each patient [93]. Analyses of this nature are optimized through the combined use of AI tools, enabling the evaluation of such extensive datasets and mitigating potential variability among different observers.

As previously discussed, the image analysis process begins with data pre-processing, which standardizes intensity, contrast, and resolution. Subsequently, relevant features are extracted to serve as inputs for ML algorithms. In supervised learning, labeled images are used to train models for tasks such as segmenting regions of interest, while unsupervised learning identifies patterns without explicit labels [94].

Convolutional Neural Networks (CNNs) are widely used in this domain due to their capacity to learn hierarchical features directly from image data. These networks consist of convolutional layers that extract local spatial features, followed by fully connected layers that integrate this information for classification or segmentation tasks. Model training typically involves the iterative adjustment of weights and biases based on the error between predicted and actual outputs, using optimization techniques such as stochastic gradient descent and backpropagation [95,96].

ML-based segmentation methods also include algorithms such as Random Forests, Support Vector Machines, and fully connected neural networks. These models learn from labeled datasets and adapt to variations in image characteristics, improving segmentation accuracy. Their performance is typically evaluated through cross-validation and metrics such as precision, recall, F1 score, and the ROC curve [97].

While these conventional approaches have shown success in various image segmentation tasks, recent advancements in foundation models have enabled more flexible and generalized solutions. Among them, the Segment Anything Model (SAM) developed by Meta AI stands out as a prompt-based, transformer-driven segmentation model capable of performing zero-shot segmentation across diverse visual domains [98].

The integration of QuPath, an open-source platform for digital pathology and biomedical image analysis [41], with SAM represents a significant advancement in automating the detection and quantification of histological structures. By incorporating the SAM extension, a transformer-based, prompt-driven segmentation model, users can leverage state-of-the-art AI to enhance segmentation accuracy and efficiency [99]. SAM is based on Vision Transformers and operates through prompt inputs, such as points, bounding boxes, or masks, but can also function in a fully automatic mode depending on the implementation [100].

Unlike task-specific models, SAM was trained on a large and diverse dataset of image-mask pairs, allowing it to generalize across various image types with minimal fine-tuning. It interprets images as sequences of embedded patches, which are processed through transformer layers to generate segmentation masks conditioned on different prompts [101]. This design enables zero-shot and few-shot generalization, making SAM especially useful in domains like histopathology, where labeled data can be scarce [102].

## 10. Challenges Integrating AI with Image Analysis

Despite the availability of AI-powered automation tools and additional software features, many recent studies still rely on manual quantification [78,103,104,105]. The transition from traditional histopathology to digital pathology involves a learning curve, as pathologists must become proficient in using new technologies and technical frameworks. Furthermore, these tools are not fully accessible to everyone, whether due to financial constraints or the specialized theoretical and technical knowledge required for their use.

Implementing ML and defining pipelines within morphometry software involves navigating a landscape that requires a comprehensive understanding of both the software’s capabilities and foundational knowledge in image processing, statistical analysis, and programming. This knowledge becomes especially crucial when customizing workflows or integrating scripts to adapt the software’s functionalities to specific research needs. Whether for automating complex analyses or enhancing the software’s functionalities, a solid grasp of these technical aspects ensures that researchers can effectively harness the software’s potential.

One of the foremost challenges lies in achieving precise segmentation of structures within images. This task can be particularly daunting in scenarios where images exhibit low contrast, overlapping structures, or variations in background intensity. Since downstream analyses rely heavily on correctly delineated regions of interest, accurate segmentation forms the cornerstone of reliable morphometric results.

Another critical aspect involves the calibration and standardization of software parameters. Ensuring that these parameters are appropriately adjusted is essential for obtaining consistent and reliable measurements. Variations or misconfigurations in calibration can lead to inconsistencies in results, affecting the validity and reproducibility of findings. Moreover, validation of results remains paramount even in automated processes. Despite advancements in automation, validating outputs is crucial, especially in cases involving complex morphometric measurements.

Although the automated component assumes a significant portion of the processes, user expertise and training remain indispensable. Some morphometry software platforms may feature steep learning curves, necessitating dedicated time and effort for users to become proficient in utilizing all available functionalities and techniques effectively. This investment in training is essential for maximizing the software’s potential and enabling accurate application in research or clinical settings.

Furthermore, the generalization of ML models used in morphometry can present challenges. While these models excel in recognizing patterns within training datasets, their ability to generalize to new, unseen datasets can be limited. This limitation can lead to reduced accuracy when applied to datasets that diverge significantly from the training data, emphasizing the importance of robust model validation and adaptation.

## 11. Perspectives

The integration of software and AI in morphometry is transforming pathology by enabling more precise and detailed image analysis through advanced segmentation and pattern recognition techniques. These developments allow us to identify and quantify morphological features with greater accuracy, opening new doors for predictive and diagnostic methods that were previously out of reach.

As we look to the future, automating morphometric analysis not only boosts efficiency, reproducibility, and consistency, qualities essential to clinical research and biomedical studies, but also drives a shift toward digital pathology. This transformation enables remote examination of high-resolution images and encourages collaboration among pathologists across the globe, fostering more unified interpretations and ultimately improving diagnostic precision.

At the same time, increased dependence on AI raises important ethical questions about the transparency of algorithms and the reliability of AI-driven diagnoses. It is vital that these technologies are implemented within a strong ethical framework and subjected to rigorous validation. Despite the growing role of automation, the expertise of pathologists and morphologists remains irreplaceable. While technology can alleviate the burden of repetitive tasks, it frees specialists to focus on deeper interpretative work. Their rich knowledge of cellular morphology and clinical context allows them to recognize subtle variations and complex patterns that machines might miss.

In summary, automated tools bring undeniable improvements in efficiency and objectivity to histological image analysis, but the interpretative skills of human experts remain crucial. The future of pathology lies in fostering a partnership between advanced technologies and the insights of experienced professionals. By maintaining this balance, we can unlock the full potential of innovation to enhance diagnostic outcomes and ensure that technology truly supports the vital work of pathology.

## Figures and Tables

**Table 1:** Image analysis software in biomedical applications.

Software	Platform	License	Key Features	Programming/ Scripting Support	Typical Applications
ImageJ/ Fiji	Windows, Mac, Linux	Open Source	Extensive plugins, ROI tools, macros, 3D Viewer, segmentation, batch processing	Java (macros), Python (via plugins), JavaScript	Histology, cell counting, morphometry, fluorescence
QuPath	Windows, Mac, Linux	Open Source	Whole-slide image analysis, cell segmentation, classification, scripting engine	Groovy, JavaScript, Python (indirect)	Digital pathology, tumor microenvironment analysis
CellProfiler	Windows, Mac, Linux	Open Source	Pipeline-based workflow, batch image processing, feature extraction	Python (via integration)	High-throughput microscopy, phenotyping, screening
HALO (Indica Labs)	Windows	Commercial	Whole-slide image analysis, AI-powered classifiers, multiplexing	Python (limited, via modules)	Immunohistochemistry, AI biomarker detection
Visiopharm	Windows	Commercial	AI/deep learning, tumor quantification, stereology, tissue classification	Python	Diagnostic pathology, spatial biology, multiplex imaging
Ilastik	Windows, Mac, Linux	Open Source	Machine learning-based segmentation and classification	Python (limited, via export)	Cell segmentation, pixel classification
ZEN (Zeiss)	Windows	Commercial (with free viewer)	Image acquisition and analysis, stitching, 3D rendering	Python (ZEN Blue scripting)	Microscopy, 3D tissue imaging
Amira/ Avizo	Windows, Mac, Linux	Commercial	3D/4D image processing, volume rendering, quantification	Python, TCL	CT/MRI-based histology, structural modeling
Aiforia	Cloud-based	Commercial (SaaS)	AI-powered WSI segmentation, automated workflows, collaborative cloud environment	API available for integration	Digital pathology, biomarker quantification, AI-assisted diagnostics

Adapted and compiled for each tool: ImageJ/Fiji [40], QuPath [41], CellProfiler 4 [42], HALO [43], Visiopharm [44], Ilastik [45], ZEN [46], Amira 5.4/Avizo 6.3 [47], and Aiforia v4.6 [48]. Note: AI, Artificial Intelligence; API, Application Programming Interface; CT, Computed Tomography; GUI, Graphical User Interface; ML, Machine Learning; MRI, Magnetic Resonance Imaging; ROI, Region of Interest; SaaS, Software as a Service; WSI, Whole-Slide Imaging.

## Data Availability

The original contributions presented in this study are included in the article/Supplementary Materials. Further inquiries can be directed to the corresponding authors.

## Supplementary Materials

The following supporting information can be downloaded at: https://www.mdpi.com/article/10.3390/jimaging12040173/s1. Data File S1: Liver morphometric analyses.
