# Supplementary material for: Cracking the Code: Computational Image Analysis Tools for Histopathological and Morphometric Insights
Source: J Imaging. 2026 Apr 17;12(4):173. doi: 10.3390/jimaging12040173 (PMC13118002; doi:10.3390/jimaging12040173)
Supplement: Supplementary file 1 [file jimaging-12-00173-s001.zip › jimaging-3822635-supplementary.pdf]

# LIVER MORPHOMETRIC ANALYSES

## HEPATOCYTE COUNTING – PART I

### Hepatocyte counting – Ten images from one sectioning plane

#### NC – Plane A

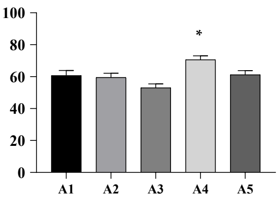


#### 24 h.p.i. – Plane A

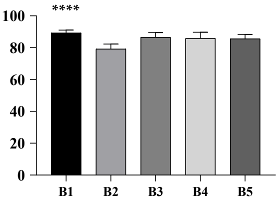


#### 48 h.p.i. – Plane A

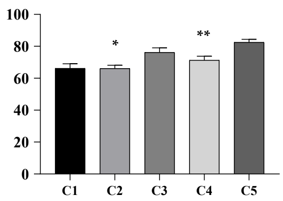


#### 72 h.p.i. – Plane A

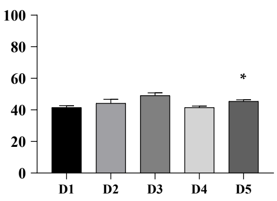


#### 96 h.p.i. – Plane A

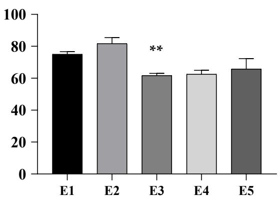


#### NC – Plane B

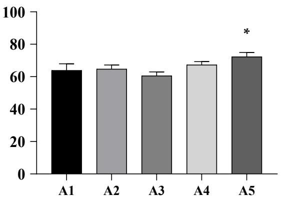


#### 24 h.p.i. – Plane B

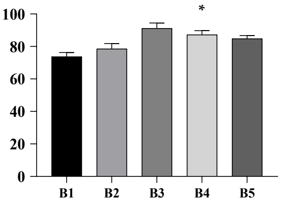


#### 48 h.p.i. – Plane B

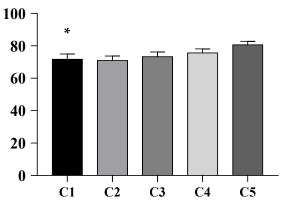


#### 72 h.p.i. – Plane B

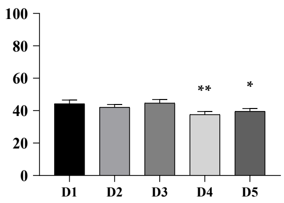


#### 96 h.p.i. – Plane B

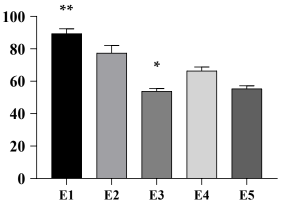


#### NC – Plane C

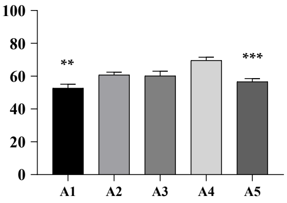


#### 24 h.p.i. – Plane C

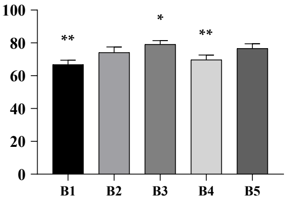


#### 48 h.p.i. – Plane C

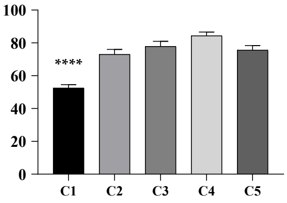


#### 72 h.p.i. – Plane C

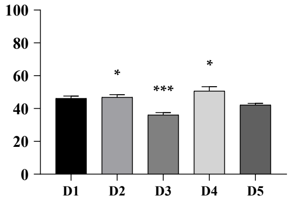


#### 96 h.p.i. – Plane C

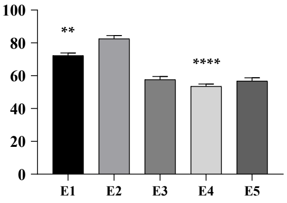


### Hepatocyte counting – Thirty images from one sectioning plane

#### NC – Plane A

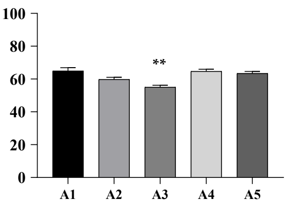


#### 24 h.p.i. – Plane A

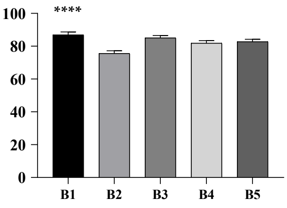


#### 48 h.p.i. – Plane A

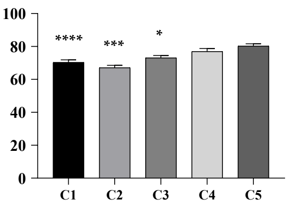


#### 72 h.p.i. – Plane A

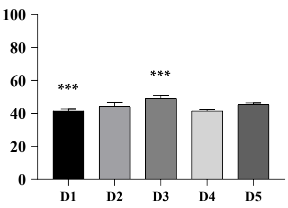


#### 96 h.p.i. – Plane A

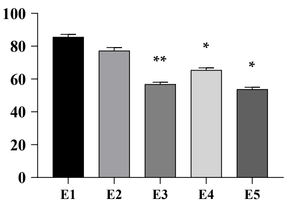


#### NC – Plane B

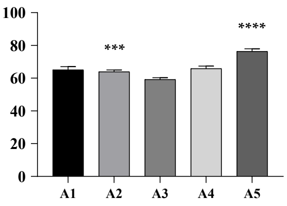


#### 24 h.p.i. – Plane B

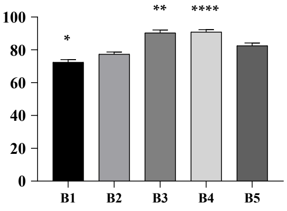


#### 48 h.p.i. – Plane B

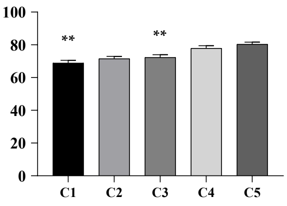


#### 72 h.p.i. – Plane B

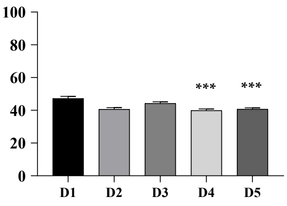


#### 96 h.p.i. – Plane B

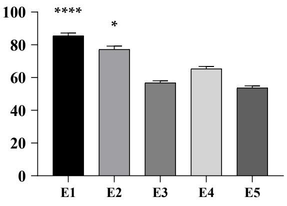


#### NC – Plane C

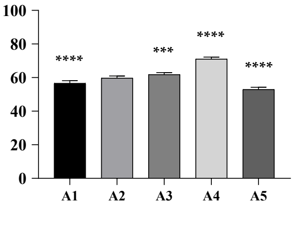


#### 24 h.p.i. – Plane C

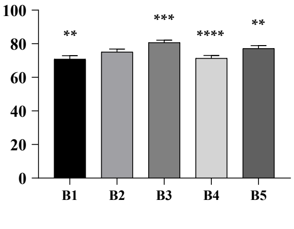


#### 48 h.p.i. – Plane C

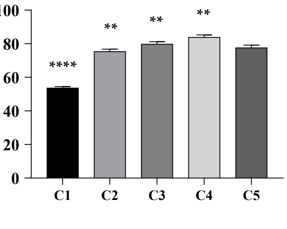


#### 72 h.p.i. – Plane C

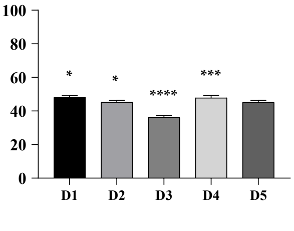


#### 96 h.p.i. – Plane C

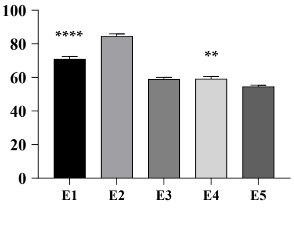


NC: Negative Control; h.p.i.: hours post-infection. Statistical analysis: unpaired t test with Welch's correction. *$p < 0.05$; **$p < 0.01$; ***$p < 0.001$; ****$p < 0.0001$.

# HEPATOCYTE COUNTING – PART II

## Hepatocyte counting – Forty images from one sectioning plane

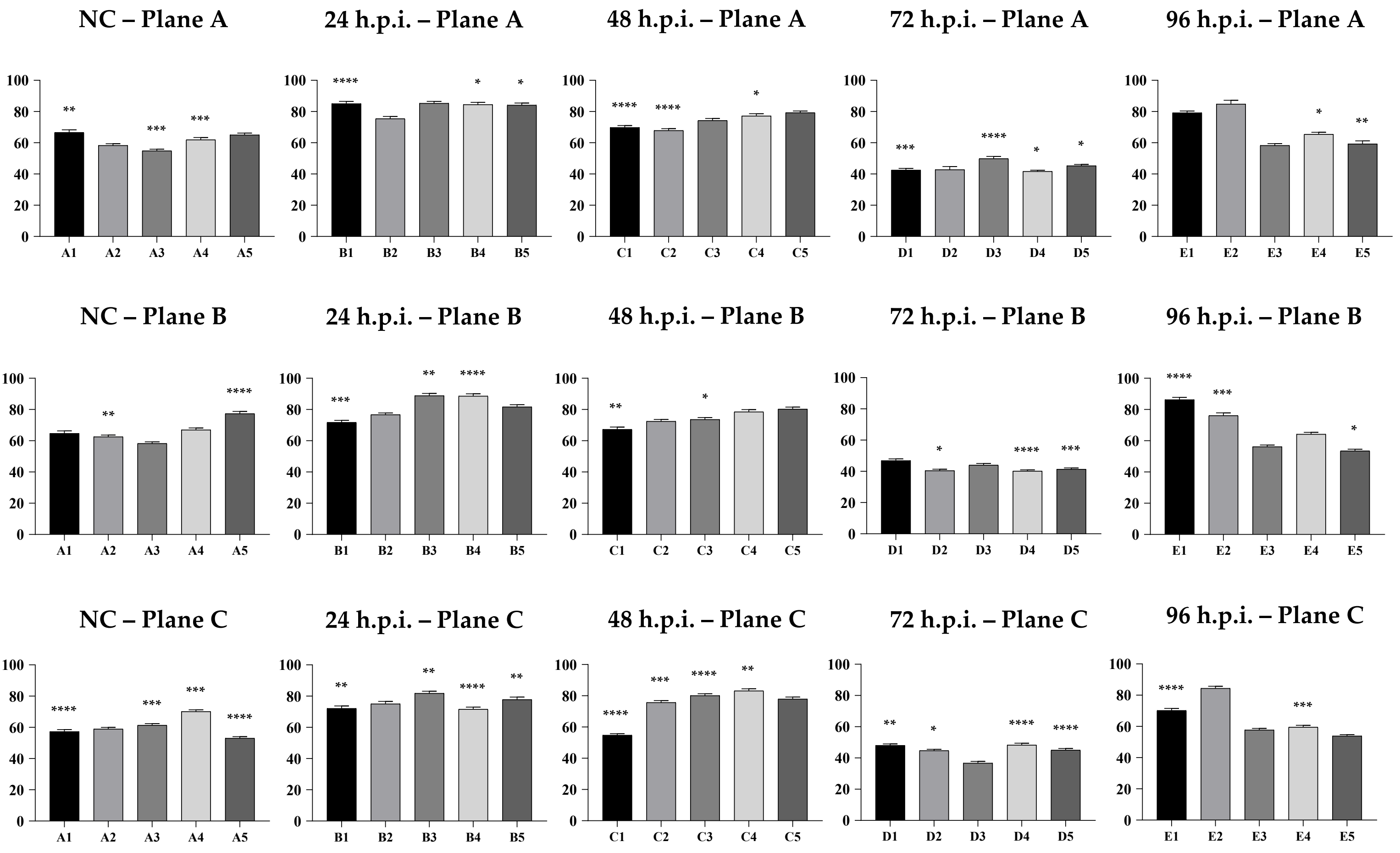


NC: Negative Control; h.p.i.: hours post-infection. Statistical analysis: unpaired t test with Welch's correction. *p < 0.05; **p < 0.01; ***p < 0.001; ****p < 0.0001.

# HEPATOCYTE COUNTING – PART III

## Hepatocyte counting – Thirty images from three sectioning planes

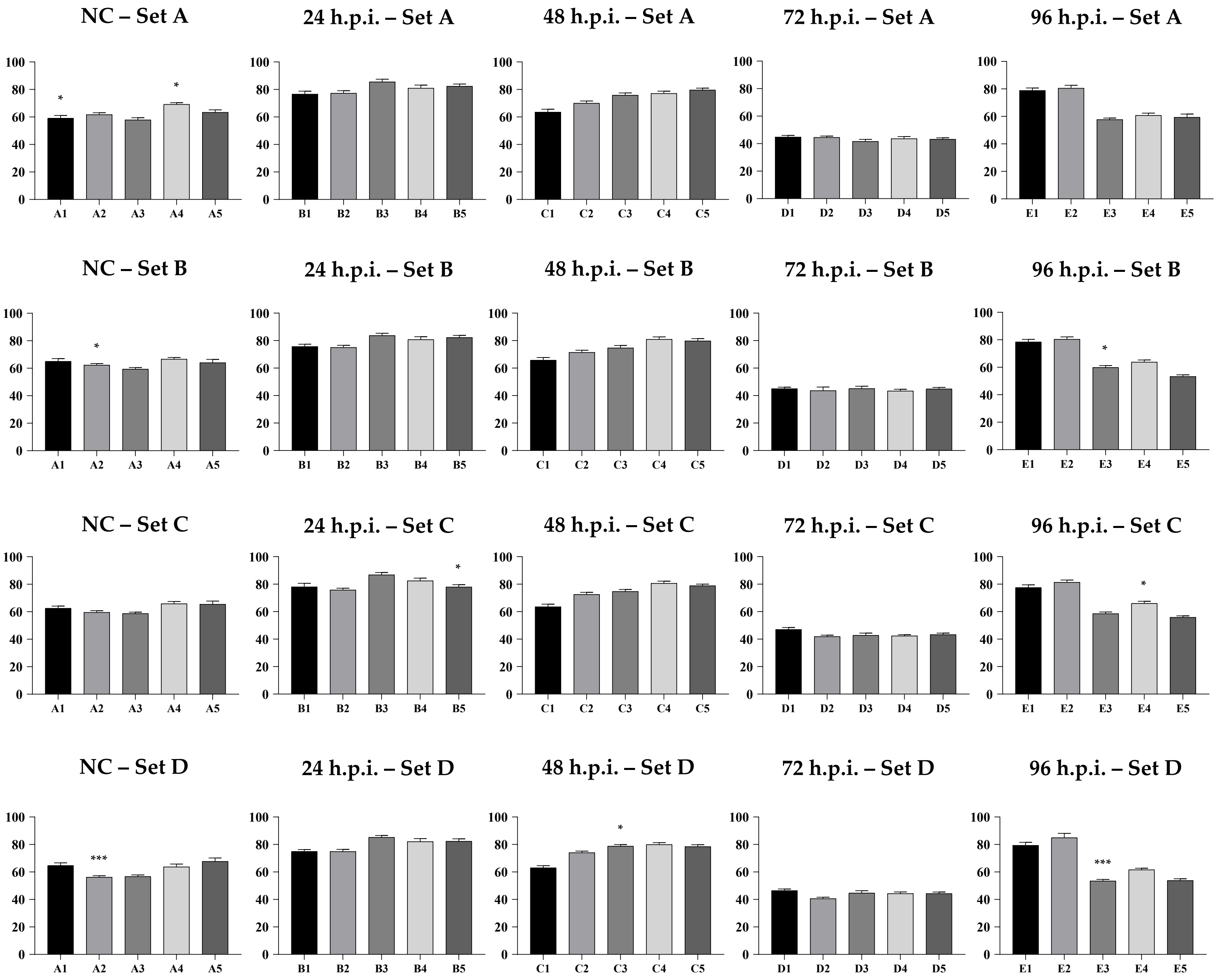


## Hepatocyte counting – Total (120 images)

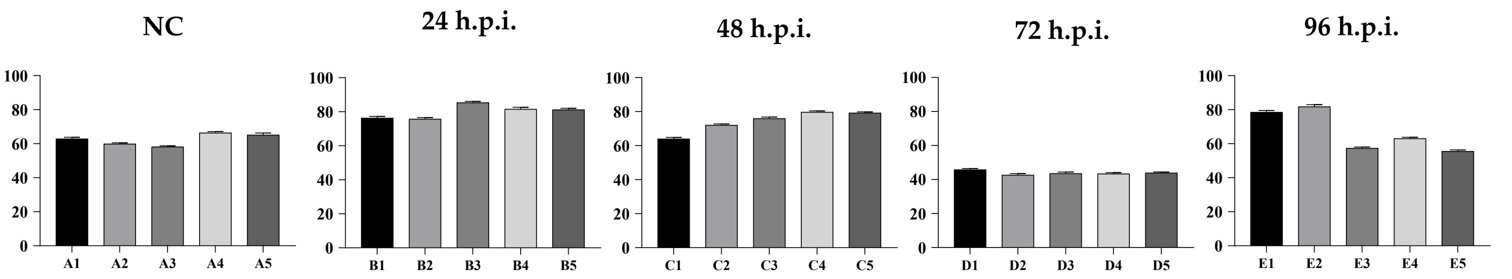


NC: Negative Control; h.p.i.: hours post-infection. Statistical analysis: unpaired t test with Welch's correction. *p < 0.05; **p < 0.01; ***p < 0.001; ****p < 0.0001.

# HEPATOCYTE COUNTING – DESCRIPTIVE STATISTICS AND COMPARATIVE ANALYSIS BY UNPAIRED T TEST WITH WELCH'S CORRECTION – NEGATIVE CONTROL – PART I

| | | | Animal 1 | Animal 2 | Animal 3 | Animal 4 | Animal 5 |
|---|---|---|---|---|---|---|---|
| Plane A | 10 Images | Subset Mean | 60.70 | 59.50 | 53.00 | 70.60 | 61.20 |
| | | (Total – Subset) Mean | 63.05 | 59.94 | 58.65 | 65.96 | 65.49 |
| | | p value | > 0.05 | > 0.05 | 0.0481 | > 0.05 | >0.05 |
| | | Mean Difference ± SEM | 2.355 ± 3.347 | 0.436 ± 2.721 | 5.645 ± 2.515 | -4.636 ± 2.565 | 4.291 ± 2.877 |
| | 30 Images | Subset Mean | 64.97 | 59.77 | 55.07 | 64.70 | 63.47 |
| | | (Total – Subset) Mean | 62.16 | 59.94 | 59.21 | 66.90 | 65.69 |
| | | p value | > 0.05 | > 0.05 | 0.0039 | > 0.05 | > 0.05 |
| | | Mean Difference ± SEM | -2.811 ± 2.176 | 0.178 ± 1.508 | 4.144 ± 1.369 | 2.200 ± 1.580 | 2.222 ± 1.879 |
| | 40 Images | Subset Mean | 66.63 | 58.25 | 54.90 | 61.90 | 65.03 |
| | | (Total – Subset) Mean | 60.98 | 60.73 | 59.81 | 68.55 | 65.19 |
| | | p value | 0.0056 | > 0.05 | 0.0001 | 0.0002 | > 0.05 |
| | | Mean Difference ± SEM | -5.650 ± 2.017 | 2.475 ± 1.391 | 4.913 ± 1.210 | 6.600 ± 1.682 | 0.1625 ± 1.989 |
| Plane B | 10 Images | Subset Mean | 63.90 | 64.70 | 60.50 | 67.30 | 72.30 |
| | | (Total – Subset) Mean | 62.76 | 59.46 | 57.96 | 66.26 | 64.48 |
| | | p value | > 0.05 | > 0.05 | > 0.05 | > 0.05 | 0.0185 |
| | | Mean Difference ± SEM | -1.136 ± 4.173 | -5.236 ± 2.622 | -2.536 ± 2.576 | -1.036 ± 2.172 | -7.818 ± 2.907 |
| | 30 Images | Subset Mean | 65.10 | 63.93 | 59.20 | 65.90 | 76.40 |
| | | (Total – Subset) Mean | 62.11 | 58.56 | 57.83 | 66.50 | 61.38 |
| | | p value | > 0.05 | 0.0002 | > 0.05 | > 0.05 | < 0.0001 |
| | | Mean Difference ± SEM | -2.989 ± 2.256 | -5.378 ± 1.348 | -1.367 ± 1.351 | 0.600 ± 1.719 | -15.02 ± 1.968 |
| | 40 Images | Subset Mean | 64.68 | 62.53 | 58.25 | 66.95 | 77.35 |
| | | (Total – Subset) Mean | 61.95 | 58.59 | 58.14 | 66.05 | 59.03 |
| | | p value | > 0.05 | 0.0041 | > 0.05 | > 0.05 | > 0.05 |
| | | Mean Difference ± SEM | -2.725 ± 2.012 | -3.938 ± 1.330 | -0.113 ± 1.301 | -0.900 ± 1.626 | -18.33 ± 1.731 |

SEM: Standard Error of the Mean.

## HEPATOCYTE COUNTING – DESCRIPTIVE STATISTICS AND COMPARATIVE ANALYSIS BY UNPAIRED T TEST WITH WELCH'S CORRECTION – NEGATIVE CONTROL – PART II

| | | | Animal 1 | Animal 2 | Animal 3 | Animal 4 | Animal 5 |
|---|---|---|---|---|---|---|---|
| Plane C | 10 Images | Subset Mean | 52.70 | 60.80 | 60.20 | 69.60 | 56.60 |
| | | (Total – Subset) Mean | 63.78 | 59.82 | 57.99 | 66.05 | 65.91 |
| | | p value | 0.0011 | > 0.05 | > 0.05 | > 0.05 | 0.0007 |
| | | Mean Difference ± SEM | 11.08 ± 2.598 | -0.982 ± 1.790 | -2.209 ± 2.874 | -3.545 ± 2.145 | 9.309 ± 2.264 |
| | 30 Images | Subset Mean | 56.63 | 59.73 | 61.77 | 71.07 | 52.97 |
| | | (Total – Subset) Mean | 64.93 | 59.96 | 56.98 | 64.78 | 69.19 |
| | | p value | < 0.0001 | > 0.05 | 0.0009 | < 0.0001 | < 0.0001 |
| | | Mean Difference ± SEM | 8.300 ± 1.844 | 0.222 ± 1.461 | -4.789 ± 1.353 | -6.289 ± 1.483 | 16.22 ± 1.694 |
| | 40 Images | Subset Mean | 57.28 | 58.93 | 61.38 | 70.15 | 53.03 |
| | | (Total – Subset) Mean | 65.65 | 60.39 | 56.58 | 64.45 | 71.19 |
| | | p value | < 0.0001 | > 0.05 | 0.0002 | 0.0002 | < 0.0001 |
| | | Mean Difference ± SEM | 8.375 ± 1.709 | 1.463 ± 1.301 | -4.800 ± 1.236 | -5.700 ± 1.450 | 18.16 ± 1.511 |
| 30 Images Multi-plane Sets | Set A | Subset Mean | 59.10 | 61.67 | 57.90 | 69.17 | 63.37 |
| | | (Total – Subset) Mean | 64.11 | 59.31 | 58.27 | 65.41 | 65.72 |
| | | p value | 0.0335 | > 0.05 | > 0.05 | 0.0182 | > 0.05 |
| | | Mean Difference ± SEM | 5.011 ± 2.286 | -2.356 ± 1.538 | 0.367 ± 1.704 | -3.756 ± 1.551 | 2.356 ± 2.290 |
| | Set B | Subset Mean | 65.03 | 62.27 | 59.40 | 66.63 | 64.00 |
| | | (Total – Subset) Mean | 62.13 | 59.11 | 57.77 | 66.26 | 65.51 |
| | | p value | > 0.05 | 0.0294 | > 0.05 | > 0.05 | > 0.05 |
| | | Mean Difference ± SEM | -2.900 ± 2.298 | -3.156 ± 1.410 | -1.633 ± 1.364 | -0.378 ± 1..560 | 1.511 ± 2.786 |
| | Set C | Subset Mean | 62.57 | 59.10 | 58.73 | 65.87 | 65.47 |
| | | (Total – Subset) Mean | 62.96 | 60.03 | 57.99 | 66.51 | 65.02 |
| | | p value | > 0.05 | > 0.05 | > 0.05 | > 0.05 | > 0.05 |
| | | Mean Difference ± SEM | 0.389 ± 1.940 | 0.534 ± 1.462 | -0.744 ± 1.241 | 0.644 ± 1.848 | -0.444 ± 2.651 |
| | Set D | Subset Mean | 64.73 | 56.17 | 56.67 | 63.73 | 67.70 |
| | | (Total – Subset) Mean | 62.23 | 61.14 | 58.68 | 67.22 | 64.28 |
| | | p value | > 0.05 | 0.0004 | > 0.05 | > 0.05 | > 0.05 |
| | | Mean Difference ± SEM | -2.500 ± 2.174 | 4.978 ± 2.198 | 2.011 ± 1.401 | 3.489 ± 2.198 | -3.422 ± 2.797 |

SEM: Standard Error of the Mean.

## HEPATOCYTE COUNTING – DESCRIPTIVE STATISTICS AND COMPARATIVE ANALYSIS 24 HOURS POST-INFECTION – PART I

| | | | Animal 1 | Animal 2 | Animal 3 | Animal 4 | Animal 5 |
|---|---|---|---|---|---|---|---|
| Plane A | 10 Images | Subset Mean | 89.30 | 79.20 | 86.50 | 85.90 | 85.60 |
| | | (Total – Subset) Mean | 75.13 | 75.42 | 85.23 | 81.17 | 80.83 |
| | | p value | < 0.0001 | > 0.05 | > 0.05 | > 0.05 | > 0.05 |
| | | Mean Difference ± SEM | -14.17 ± 2.027 | -3.782 ± 3.205 | -1.273 ± 3.079 | -4.727 ± 3.942 | -4.773 ± 2.925 |
| | 30 Images | Subset Mean | 86.93 | 75.60 | 85.10 | 81.90 | 82.77 |
| | | (Total – Subset) Mean | 72.77 | 75.78 | 85.41 | 81.46 | 80.71 |
| | | p value | < 0.0001 | > 0.05 | > 0.05 | > 0.05 | > 0.05 |
| | | Mean Difference ± SEM | -14.17 ± 1.992 | 0.1778 ± 1.888 | 0.3111 ± 1.727 | -0.444± 1.984 | -2.056 ± 1.778 |
| | 40 Images | Subset Mean | 85.00 | 75.48 | 85.30 | 84.48 | 84.13 |
| | | (Total – Subset) Mean | 71.96 | 75.86 | 85.35 | 80.11 | 79.78 |
| | | p value | < 0.0001 | > 0.05 | > 0.05 | 0.0307 | 0.0119 |
| | | Mean Difference ± SEM | -13.04 ± 1.816 | 0.3875 ± 1.715 | 0.050 ± 1.608 | -4.363 ± 1.991 | -4.350 ± 1.691 |
| Plane B | 10 Images | Subset Mean | 73.70 | 78.50 | 91.10 | 87.20 | 84.80 |
| | | (Total – Subset) Mean | 76.55 | 75.48 | 84.81 | 81.05 | 80.90 |
| | | p value | > 0.05 | > 0.05 | > 0.05 | 0.0408 | > 0.05 |
| | | Mean Difference ± SEM | 2.845 ± 2.822 | -3.018 ± 3.378 | -6.291 ± 3.469 | -6.145 ± 2.707 | -3.900 ± 2.099 |
| | 30 Images | Subset Mean | 72.47 | 77.37 | 90.33 | 90.87 | 82.53 |
| | | (Total – Subset) Mean | 77.59 | 75.19 | 83.67 | 78.47 | 80.79 |
| | | p value | 0.0122 | > 0.05 | 0.0015 | < 0.0001 | > 0.05 |
| | | Mean Difference ± SEM | 5.122 ± 1.988 | -2.178 ± 1.638 | -6.667 ± 1.963 | -12.40 ± 1.803 | -1.744 ± 1.908 |
| | 40 Images | Subset Mean | 71.80 | 76.70 | 88.88 | 88.60 | 81.73 |
| | | (Total – Subset) Mean | 78.56 | 75.25 | 83.56 | 78.05 | 80.98 |
| | | p value | 0.0003 | > 0.05 | 0.0026 | < 0.0001 | > 0.05 |
| | | Mean Difference ± SEM | 6.763 ± 1.825 | -1.450 ± 1.532 | -5.313 ± 1.699 | -10.55 ± 1.919 | -0.750 ± 1.689 |

SEM: Standard Error of the Mean.

# HEPATOCYTE COUNTING – DESCRIPTIVE STATISTICS AND COMPARATIVE ANALYSIS 24 HOURS POST-INFECTION – PART II

| | | | Animal 1 | Animal 2 | Animal 3 | Animal 4 | Animal 5 |
|---|---|---|---|---|---|---|---|
| Plane C | 10 Images | Subset Mean | 66.80 | 74.10 | 79.10 | 69.70 | 76.60 |
| | | (Total – Subset) Mean | 77.17 | 75.88 | 85.90 | 82.65 | 81.65 |
| | | p value | 0.0034 | > 0.05 | 0.0177 | 0.0005 | 0.0015 |
| | | Mean Difference ± SEM | 10.37 ± 2.846 | 1.782 ± 3.523 | 6.800 ± 2.456 | 12.95 ± 3.122 | 5.045 ± 2.955 |
| | 30 Images | Subset Mean | 70.90 | 75.10 | 80.67 | 71.37 | 77.23 |
| | | (Total – Subset) Mean | 78.11 | 75.94 | 86.89 | 84.97 | 82.56 |
| | | p value | 0.0025 | > 0.05 | 0.005 | < 0.0001 | 0.0062 |
| | | Mean Difference ± SEM | 7.211 ± 2.264 | 0.844 ± 2.017 | 6.222 ± 1.684 | 13.60 ± 1.996 | 5.322 ± 1.863 |
| | 40 Images | Subset Mean | 72.13 | 75.03 | 81.83 | 71.63 | 77.83 |
| | | (Total – Subset) Mean | 78.40 | 76.09 | 87.09 | 86.54 | 82.93 |
| | | p value | 0.0026 | > 0.05 | 0.0016 | < 0.0001 | < 0.0001 |
| | | Mean Difference ± SEM | 6.275 ± 2.024 | 1.063 ± 1.849 | 5.263 ± 1.608 | 14.91 ± 1.724 | 5.100 ± 1.790 |
| 30 Images Multi-plane Sets | Set A | Subset Mean | 76.60 | 77.27 | 85.57 | 80.93 | 82.33 |
| | | (Total – Subset) Mean | 76.21 | 75.22 | 85.26 | 81.78 | 80.86 |
| | | p value | > 0.05 | > 0.05 | > 0.05 | > 0.05 | > 0.05 |
| | | Mean Difference ± SEM | -0.389 ± 2.471 | -2.044 ± 2.060 | -0.311 ± 2.061 | 0.844 ± 2.569 | -1.478 ± 1.879 |
| | Set B | Subset Mean | 75.67 | 75.00 | 83.73 | 80.77 | 82.27 |
| | | (Total – Subset) Mean | 76.52 | 75.98 | 85.87 | 81.83 | 80.88 |
| | | p value | > 0.05 | > 0.05 | > 0.05 | > 0.05 | > 0.05 |
| | | Mean Difference ± SEM | 0.8556 ± 2.133 | 0.9778 ± 1.898 | 2.133 ± 1.853 | 1.067 ± 2.422 | -1.389 ± 1.811 |
| | Set C | Subset Mean | 74.93 | 74.87 | 85.23 | 82.13 | 82.37 |
| | | (Total – Subset) Mean | 76.77 | 76.02 | 85.37 | 81.38 | 80.84 |
| | | p value | > 0.05 | > 0.05 | > 0.05 | > 0.05 | > 0.05 |
| | | Mean Difference ± SEM | 1.833 ± 1.884 | 1.156 ± 1.864 | 0.133 ± 1.617 | -0.756 ± 2.439 | -1.522 ± 2.021 |
| | Set D | Subset Mean | 74.93 | 74.87 | 85.23 | 82.13 | 82.37 |
| | | (Total – Subset) Mean | 76.77 | 76.02 | 85.37 | 81.38 | 80.84 |
| | | p value | > 0.05 | > 0.05 | > 0.05 | > 0.05 | > 0.05 |
| | | Mean Difference ± SEM | 1.833 ± 1.884 | 1.156 ± 1.864 | 0.133 ± 1.617 | -0.756 ± 2.439 | -1.522±2.021 |

SEM: Standard Error of the Mean.

# HEPATOCYTE COUNTING – DESCRIPTIVE STATISTICS AND COMPARATIVE ANALYSIS 48 HOURS POST-INFECTION– PART I

| | | | Animal 1 | Animal 2 | Animal 3 | Animal 4 | Animal 5 |
|---|---|---|---|---|---|---|---|
| Plane A | 10 Images | Subset Mean | 66.20 | 66.10 | 76.20 | 71.30 | 82.50 |
| | | (Total – Subset) Mean | 63.75 | 72.55 | 75.97 | 80.43 | 78.87 |
| | | p value | > 0.05 | 0.0109 | > 0.05 | 0.0053 | > 0.05 |
| | | Mean Difference ± SEM | -2.445 ± 3.017 | 6.455 ± 2.137 | -0.227 ± 2.914 | 9.127 ± 2.641 | -3.627 ± 2.024 |
| | 30 Images | Subset Mean | 70.33 | 67.10 | 73.13 | 76.97 | 80.27 |
| | | (Total – Subset) Mean | 61.83 | 73.66 | 76.94 | 80.57 | 78.81 |
| | | p value | < 0.0001 | < 0.0001 | 0.0266 | > 0.05 | > 0.05 |
| | | Mean Difference ± SEM | -8.500 ± 1.854 | 6.556 ± 1.662 | 3.811 ± 1.670 | 3.600 ± 2.016 | -1.456 ± 1.585 |
| | 40 Images | Subset Mean | 69.78 | 67.90 | 74.25 | 77.23 | 79.25 |
| | | (Total – Subset) Mean | 61.05 | 74.08 | 76.86 | 80.89 | 79.14 |
| | | p value | < 0.0001 | < 0.0001 | > 0.05 | 0.0338 | > 0.05 |
| | | Mean Difference ± SEM | -8.725 ± 1.686 | 6.175 ± 1.442 | 2.613 ± 1.578 | 3.663 ± 1.695 | -0.113 ± 1.422 |
| Plane B | 10 Images | Subset Mean | 71.80 | 71.00 | 73.40 | 75.70 | 80.70 |
| | | (Total – Subset) Mean | 63.25 | 72.11 | 76.23 | 80.03 | 79.04 |
| | | p value | 0.0246 | > 0.05 | > 0.05 | > 0.05 | > 0.05 |
| | | Mean Difference ± SEM | -8.555 ± 3.275 | 1.109 ± 2.814 | 2.827 ± 2.949 | 4.327 ± 2.604 | -1.664 ± 2.232 |
| | 30 Images | Subset Mean | 68.83 | 71.53 | 72.30 | 77.87 | 80.33 |
| | | (Total – Subset) Mean | 62.33 | 72.18 | 72.22 | 80.27 | 78.79 |
| | | p value | 0.0018 | > 0.05 | 0.0095 | > 0.05 | > 0.05 |
| | | Mean Difference ± SEM | -6.500 ± 1.978 | 0.644 ± 1.654 | 4.922 ± 1.816 | 2.400 ± 1.845 | -1.544 ± 1.502 |
| | 40 Images | Subset Mean | 67.33 | 72.43 | 73.60 | 78.58 | 80.33 |
| | | (Total – Subset) Mean | 62.28 | 71.81 | 77.19 | 80.21 | 78.60 |
| | | p value | 0.0082 | > 0.05 | 0.0261 | > 0.05 | > 0.05 |
| | | Mean Difference ± SEM | -5.050 ± 1.865 | -0.613 ± 1.497 | 3.588 ± 1.582 | 1.638 ± 1.718 | -1.725 ± 1.461 |

SEM: Standard Error of the Mean.

# HEPATOCYTE COUNTING – DESCRIPTIVE STATISTICS AND COMPARATIVE ANALYSIS 48 HOURS POST-INFECTION – PART II

| | | | Animal 1 | Animal 2 | Animal 3 | Animal 4 | Animal 5 |
|---|---|---|---|---|---|---|---|
| Plane C | 10 Images | Subset Mean | 52.50 | 73.00 | 77.90 | 84.40 | 75.60 |
| | | (Total – Subset) Mean | 65.00 | 71.93 | 75.82 | 79.24 | 79.50 |
| | | p value | < 0.0001 | > 0.05 | > 0.05 | > 0.05 | > 0.05 |
| | | Mean Difference ± SEM | 12.50 ± 2.285 | -1.073 ± 3.151 | -2.082 ± 3.139 | -5.164 ± 2.368 | 3.900 ± 2.828 |
| | 30 Images | Subset Mean | 53.67 | 75.40 | 79.77 | 83.90 | 77.63 |
| | | (Total – Subset) Mean | 67.39 | 70.89 | 74.73 | 78.26 | 79.69 |
| | | p value | < 0.0001 | 0.0081 | 0.0039 | 0.0011 | > 0.05 |
| | | Mean Difference ± SEM | 13.72 ± 1.324 | -4.511 ± 1.636 | -5.033 ± 1.668 | -5.644 ± 1.646 | 2.056 ± 1.745 |
| | 40 Images | Subset Mean | 54.78 | 75.73 | 80.13 | 83.20 | 77.95 |
| | | (Total – Subset) Mean | 68.55 | 70.16 | 73.93 | 77.90 | 79.79 |
| | | p value | < 0.0001 | 0.0003 | < 0.0001 | 0.0011 | > 0.05 |
| | | Mean Difference ± SEM | 13.78 ± 1.358 | -5.563 ± 1.471 | -6.200 ± 1.481 | -5.300 ± 1.571 | 1.837 ± 1.519 |
| 30 Images Multi-plane Sets | Set A | Subset Mean | 63.50 | 70.03 | 75.83 | 77.13 | 79.60 |
| | | (Total – Subset) Mean | 64.11 | 72.68 | 76.04 | 80.51 | 79.03 |
| | | p value | > 0.05 | > 0.05 | > 0.05 | > 0.05 | > 0.05 |
| | | Mean Difference ± SEM | 0.611 ± 2.372 | 2.644 ± 1.763 | 0.211 ± 1.860 | 3.378 ± 1.904 | -0.567 ± 1.595 |
| | Set B | Subset Mean | 65.80 | 71.43 | 74.67 | 80.97 | 79.80 |
| | | (Total – Subset) Mean | 67.34 | 72.21 | 76.43 | 79.23 | 78.97 |
| | | p value | > 0.05 | > 0.05 | > 0.05 | > 0.05 | > 0.05 |
| | | Mean Difference ± SEM | -2.456 ± 2.160 | 0.778 ± 1.819 | 1.767 ± 1.955 | -1.733 ± 1.966 | -0.833 ± 1.797 |
| | Set C | Subset Mean | 63.53 | 72.57 | 74.70 | 80.63 | 78.83 |
| | | (Total – Subset) Mean | 64.10 | 71.83 | 76.42 | 79.34 | 79.29 |
| | | p value | > 0.05 | > 0.05 | > 0.05 | > 0.05 | > 0.05 |
| | | Mean Difference ± SEM | 0.567 ± 2.183 | -0.733 ± 1.696 | 1.722 ± 1.659 | -1.289 ± 1.838 | 0.456 ± 1.480 |
| | Set D | Subset Mean | 63.00 | 74.03 | 78.77 | 79.93 | 78.47 |
| | | (Total – Subset) Mean | 64.28 | 71.34 | 75.07 | 79.58 | 79.41 |
| | | p value | > 0.05 | > 0.05 | 0.0149 | > 0.05 | > 0.05 |
| | | Mean Difference ± SEM | 1.278 ± 1.927 | -2.689 ± 1.461 | -3.700 ± 1.481 | -0.356 ± 1.703 | 0.944 ± 1.554 |

SEM: Standard Error of the Mean.

## HEPATOCYTE COUNTING – DESCRIPTIVE STATISTICS AND COMPARATIVE ANALYSIS 72 HOURS POST-INFECTION – PART I

| | | | Animal 1 | Animal 2 | Animal 3 | Animal 4 | Animal 5 |
|---|---|---|---|---|---|---|---|
| Plane A | 10 Images | Subset Mean | 43.80 | 44.60 | 44.10 | 42.50 | 47.70 |
| | | (Total – Subset) Mean | 46.00 | 42.47 | 43.51 | 43.47 | 43.55 |
| | | p value | > 0.05 | > 0.05 | > 0.05 | > 0.05 | 0.0246 |
| | | Mean Difference ± SEM | 2.200 ± 2.676 | -2.127 ± 2.143 | -0.591 ± 3.293 | 0.973 ±1.927 | -4.415 ± 1.605 |
| | 30 Images | Subset Mean | 41.57 | 44.17 | 49.10 | 41.57 | 45.47 |
| | | (Total – Subset) Mean | 46.91 | 42.33 | 41.92 | 43.76 | 43.61 |
| | | p value | 0.0002 | > 0.05 | 0.0005 | > 0.05 | > 0.05 |
| | | Mean Difference ± SEM | 5.347 ± 1.316 | -1.833 ± 2.657 | -7.175 ± 1.910 | 2.197 ± 1.183 | -1.854 ± 1.124 |
| | 40 Images | Subset Mean | 42.55 | 42.80 | 49.90 | 41.68 | 45.33 |
| | | (Total – Subset) Mean | 47.45 | 42.58 | 40.39 | 44.25 | 43.19 |
| | | p value | 0.0002 | > 0.05 | < 0.0001 | 0.0283 | 0.0453 |
| | | Mean Difference ± SEM | 4.900 ± 1.273 | -0.225 ± 2.116 | -9.513 ± 1.591 | 2.575 ± 1.158 | -2.138 ± 1.053 |
| Plane B | 10 Images | Subset Mean | 44.30 | 42.10 | 44.70 | 37.60 | 39.60 |
| | | (Total – Subset) Mean | 45.95 | 42.70 | 43.45 | 43.92 | 44.29 |
| | | p value | > 0.05 | > 0.05 | > 0.05 | 0.0089 | 0.0249 |
| | | Mean Difference ± SEM | 1.655 ± 2.372 | 0.600 ± 1.882 | -1.245 ± 2.372 | 6.318 ± 1.998 | 4.691 ± 1.806 |
| | 30 Images | Subset Mean | 47.17 | 40.60 | 44.17 | 39.90 | 40.70 |
| | | (Total – Subset) Mean | 45.37 | 43.33 | 43.36 | 44.56 | 44.97 |
| | | p value | > 0.05 | > 0.05 | > 0.05 | 0.0004 | 0.0001 |
| | | Mean Difference ± SEM | -1.800 ± 1.535 | 2.733 ± 1.423 | -0.811 ± 1.481 | 4.656 ± 1.236 | 4.267 ± 1.053 |
| | 40 Images | Subset Mean | 46.88 | 40.48 | 44.05 | 40.20 | 41.35 |
| | | (Total – Subset) Mean | 45.29 | 43.74 | 43.31 | 44.99 | 45.18 |
| | | p value | > 0.05 | 0.0214 | > 0.05 | < 0.0001 | 0.0005 |
| | | Mean Difference ± SEM | -1.588 ± 1.312 | 3.263 ± 1.399 | -0.738 ± 1.482 | 4.787 ± 1.124 | 3.825 ± 1.055 |

SEM: Standard Error of the Mean.

## HEPATOCYTE COUNTING – DESCRIPTIVE STATISTICS AND COMPARATIVE ANALYSIS 72 HOURS POST-INFECTION – PART II

| | | | Animal 1 | Animal 2 | Animal 3 | Animal 4 | Animal 5 |
|---|---|---|---|---|---|---|---|
| Plane C | 10 Images | Subset Mean | 46.20 | 46.90 | 36.10 | 50.70 | 42.20 |
| | | (Total – Subset) Mean | 45.78 | 42.26 | 44.24 | 42.73 | 44.05 |
| | | p value | > 0.05 | 0.0176 | 0.0001 | 0.0139 | > 0.05 |
| | | Mean Difference ± SEM | -0.418 ± 1.599 | -4.636 ± 1.744 | 8.136 ± 1.662 | -7.973 ± 2.677 | 1.855 ± 1.102 |
| | 30 Images | Subset Mean | 48.07 | 45.23 | 36.23 | 47.80 | 45.13 |
| | | (Total – Subset) Mean | 45.07 | 41.79 | 46.00 | 41.92 | 43.49 |
| | | p value | 0.0207 | 0.0176 | < 0.0001 | 0.0004 | > 0.05 |
| | | Mean Difference ± SEM | -3.000±1.264 | -3.444 ± 1.423 | 9.767 ± 1.409 | -5.878 ± 1.522 | -1.644 ± 1.314 |
| | 40 Images | Subset Mean | 48.03 | 44.68 | 36.73 | 48.30 | 45.03 |
| | | (Total – Subset) Mean | 44.71 | 41.64 | 46.98 | 40.94 | 43.34 |
| | | p value | 0.0089 | 0.0322 | < 0.0001 | < 0.0001 | > 0.05 |
| | | Mean Difference ± SEM | -3.313 ± 1.237 | -3.037 ± 1.401 | 10.25 ± 1.384 | -7.363 ± 1.291 | -1.688 ± 1.181 |
| 30 Images Multi-plane Sets | Set A | Subset Mean | 44.77 | 44.53 | 41.63 | 43.60 | 43.17 |
| | | (Total – Subset) Mean | 46.17 | 42.02 | 44.20 | 43.32 | 44.14 |
| | | p value | > 0.05 | > 0.05 | > 0.05 | > 0.05 | > 0.05 |
| | | Mean Difference ± SEM | 1.400 ± 1.422 | -2.511 ± 1.435 | 2.567 ± 1.794 | -0.278 ± 1.689 | 0.9778 ± 1.192 |
| | Set B | Subset Mean | 45.03 | 43.63 | 45.10 | 43.33 | 44.87 |
| | | (Total – Subset) Mean | 46.08 | 42.32 | 43.04 | 43.41 | 43.58 |
| | | p value | > 0.05 | > 0.05 | > 0.05 | > 0.05 | > 0.05 |
| | | Mean Difference ± SEM | 1.044 ± 1.329 | -1.311 ± 2.687 | -2.056 ± 1.953 | 0.078 ± 1.483 | -1.289 ± 1.264 |
| | Set C | Subset Mean | 47.00 | 41.83 | 42.77 | 42.33 | 43.27 |
| | | (Total – Subset) Mean | 45.42 | 42.92 | 43.82 | 43.74 | 44.11 |
| | | p value | > 0.05 | > 0.05 | > 0.05 | > 0.05 | > 0.05 |
| | | Mean Difference ± SEM | -1.578 ± 1.631 | 1.089 ± 1.457 | 1.056 ± 1.897 | 1.411 ± 1.188 | 0.844 ± 1.233 |
| | Set D | Subset Mean | 46.47 | 40.60 | 40.60 | 44.73 | 44.30 |
| | | (Total – Subset) Mean | 45.60 | 43.33 | 43.33 | 43.17 | 43.09 |
| | | p value | > 0.05 | > 0.05 | > 0.05 | > 0.05 | > 0.05 |
| | | Mean Difference ± SEM | -0.867 ± 1.396 | 2.733 ± 1.426 | 2.733 ± 1.426 | -1.567 ± 1.910 | -1.211 ± 1.403 |

SEM: Standard Error of the Mean.

# HEPATOCYTE COUNTING – DESCRIPTIVE STATISTICS AND COMPARATIVE ANALYSIS 96 HOURS POST-INFECTION – PART I

| | | | Animal 1 | Animal 2 | Animal 3 | Animal 4 | Animal 5 |
|---|---|---|---|---|---|---|---|
| Plane A | 10 Images | Subset Mean | 75.00 | 81.70 | 61.70 | 62.50 | 65.80 |
| | | (Total – Subset) Mean | 78.89 | 81.80 | 57.01 | 63.11 | 54.64 |
| | | p value | > 0.05 | > 0.05 | 0.0085 | > 0.05 | > 0.05 |
| | | Mean Difference ± SEM | 3.891 ± 1.957 | 0.100 ± 3.944 | -4.691 ± 1.525 | 0.609 ± 2.602 | -11.16 ± 6.487 |
| | 30 Images | Subset Mean | 78.53 | 80.67 | 60.50 | 66.10 | 60.37 |
| | | (Total – Subset) Mean | 78.58 | 82.17 | 56.37 | 62.04 | 53.97 |
| | | p value | > 0.05 | > 0.05 | 0.0025 | 0.0293 | 0.0138 |
| | | Mean Difference ± SEM | 0.044 ± 1.813 | 1.500 ± 2.232 | -4.133 ± 1.307 | -4.056 ± 1.799 | -6.400 ± 2.463 |
| | 40 Images | Subset Mean | 79.23 | 84.83 | 58.33 | 65.43 | 59.28 |
| | | (Total – Subset) Mean | 78.24 | 80.28 | 56.94 | 61.88 | 53.71 |
| | | p value | > 0.05 | > 0.05 | > 0.05 | 0.0229 | 0.0088 |
| | | Mean Difference ± SEM | -0.988 ± 1.789 | -4.550 ± 2.671 | -1.388 ± 1.375 | -3.550 ± 1.529 | -5.563 ± 2.037 |
| Plane B | 10 Images | Subset Mean | 89.40 | 77.40 | 53.80 | 66.40 | 55.40 |
| | | (Total – Subset) Mean | 77.58 | 82.19 | 57.73 | 62.75 | 55.58 |
| | | p value | 0.0032 | > 0.05 | 0.0466 | > 0.05 | > 0.05 |
| | | Mean Difference ± SEM | -11.82 ± 3.152 | 4.791 ± 4.835 | 3.927 ± 1.772 | -3.645 ± 2.492 | 0.182 ± 1.981 |
| | 30 Images | Subset Mean | 85.50 | 77.20 | 56.77 | 65.40 | 53.67 |
| | | (Total – Subset) Mean | 76.26 | 83.32 | 57.61 | 62.28 | 56.20 |
| | | p value | < 0.0001 | 0.0134 | > 0.05 | > 0.05 | > 0.05 |
| | | Mean Difference ± SEM | -9.244 ± 2.033 | 6.122 ± 2.396 | 0.844 ± 1.471 | -3.122 ± 1.602 | 2.533 ± 1.613 |
| | 40 Images | Subset Mean | 86.30 | 76.15 | 56.18 | 64.20 | 53.53 |
| | | (Total – Subset) Mean | 74.70 | 84.61 | 58.01 | 62.49 | 56.59 |
| | | p value | < 0.0001 | 0.0002 | > 0.05 | > 0.05 | 0.0464 |
| | | Mean Difference ± SEM | -11.60 ± 1.819 | 8.462 ± 2.167 | 1.838 ± 1.322 | -1.713 ± 1.512 | 3.063 ± 1.520 |

SEM: Standard Error of the Mean.

## HEPATOCYTE COUNTING – DESCRIPTIVE STATISTICS AND COMPARATIVE ANALYSIS 96 HOURS POST-INFECTION – PART II

| | | | Animal 1 | Animal 2 | Animal 3 | Animal 4 | Animal 5 |
|---|---|---|---|---|---|---|---|
| Plane C | 10 Images | Subset Mean | 72.30 | 82.50 | 57.60 | 53.50 | 56.80 |
| | | (Total – Subset) Mean | 79.14 | 81.73 | 57.38 | 63.93 | 55.45 |
| | | p value | 0.0021 | > 0.05 | > 0.05 | < 0.0001 | > 0.05 |
| | | Mean Difference ± SEM | 6.836 ± 1.911 | -0.773 ± 2.313 | -0.218 ± 2.049 | 10.43 ± 1.655 | -1.345 ± 2.092 |
| | 30 Images | Subset Mean | 70.83 | 84.33 | 58.87 | 59.10 | 54.43 |
| | | (Total – Subset) Mean | 81.14 | 80.94 | 56.91 | 64.38 | 55.94 |
| | | p value | < 0.0001 | > 0.05 | > 0.05 | 0.0021 | 0.2971 |
| | | Mean Difference ± SEM | 10.31 ± 1.942 | -3.389 ± 2.113 | -1.956 ± 1.391 | 5.278 ± 1.629 | 1.511 ± 1.441 |
| | 40 Images | Subset Mean | 70.18 | 84.40 | 57.70 | 59.55 | 53.90 |
| | | (Total – Subset) Mean | 82.76 | 80.49 | 57.25 | 64.81 | 56.40 |
| | | p value | < 0.0001 | > 0.05 | > 0.05 | 0.0005 | > 0.05 |
| | | Mean Difference ± SEM | 12.59 ± 1.712 | -3.913 ± 2.024 | -0.450 ± 1.284 | 5.263 ± 1.449 | 2.500 ± 1.405 |
| 30 Images Multi-plane Sets | Set A | Subset Mean | 78.90 | 80.53 | 57.70 | 60.80 | 59.33 |
| | | (Total – Subset) Mean | 78.46 | 82.21 | 57.30 | 63.81 | 54.31 |
| | | p value | > 0.05 | > 0.05 | > 0.05 | > 0.05 | > 0.05 |
| | | Mean Difference ± SEM | -0.444 ± 2.183 | 1.678 ± 2.469 | -0.400 ± 1.337 | 3.011 ± 1.764 | -5.022 ± 2.496 |
| | Set B | Subset Mean | 78.43 | 80.33 | 59.90 | 63.80 | 53.27 |
| | | (Total – Subset) Mean | 78.61 | 82.28 | 56.57 | 62.81 | 56.33 |
| | | p value | > 0.05 | > 0.05 | 0.0290 | > 0.05 | > 0.05 |
| | | Mean Difference ± SEM | 0.178 ± 2.206 | 1.944 ± 2.245 | -3.333 ± 1.479 | -0.989 ± 1.685 | 3.067 ± 1.620 |
| | Set C | Subset Mean | 77.53 | 81.33 | 58.53 | 66.00 | 55.87 |
| | | (Total – Subset) Mean | 78.91 | 81.94 | 57.02 | 62.08 | 55.47 |
| | | p value | > 0.05 | > 0.05 | > 0.05 | 0.0304 | > 0.05 |
| | | Mean Difference ± SEM | 1.378 ± 2.318 | 0.611 ± 2.222 | -1.511 ± 1.411 | -3.922 ± 1.755 | -0.400 ± 1.479 |
| | Set D | Subset Mean | 79.40 | 84.97 | 53.47 | 61.63 | 53.80 |
| | | (Total – Subset) Mean | 78.29 | 80.73 | 58.71 | 63.53 | 56.16 |
| | | p value | > 0.05 | > 0.05 | 0.0002 | > 0.05 | > 0.05 |
| | | Mean Difference ± SEM | -1.111 ± 2.437 | -4.233 ± 3.312 | 5.244 ± 1.317 | 1.900 ± 1.402 | 2.356 ± 1.587 |

# HEPATOCYTE BINUCLEATION – PART I

## Hepatocyte binucleation – Ten images from one sectioning plane

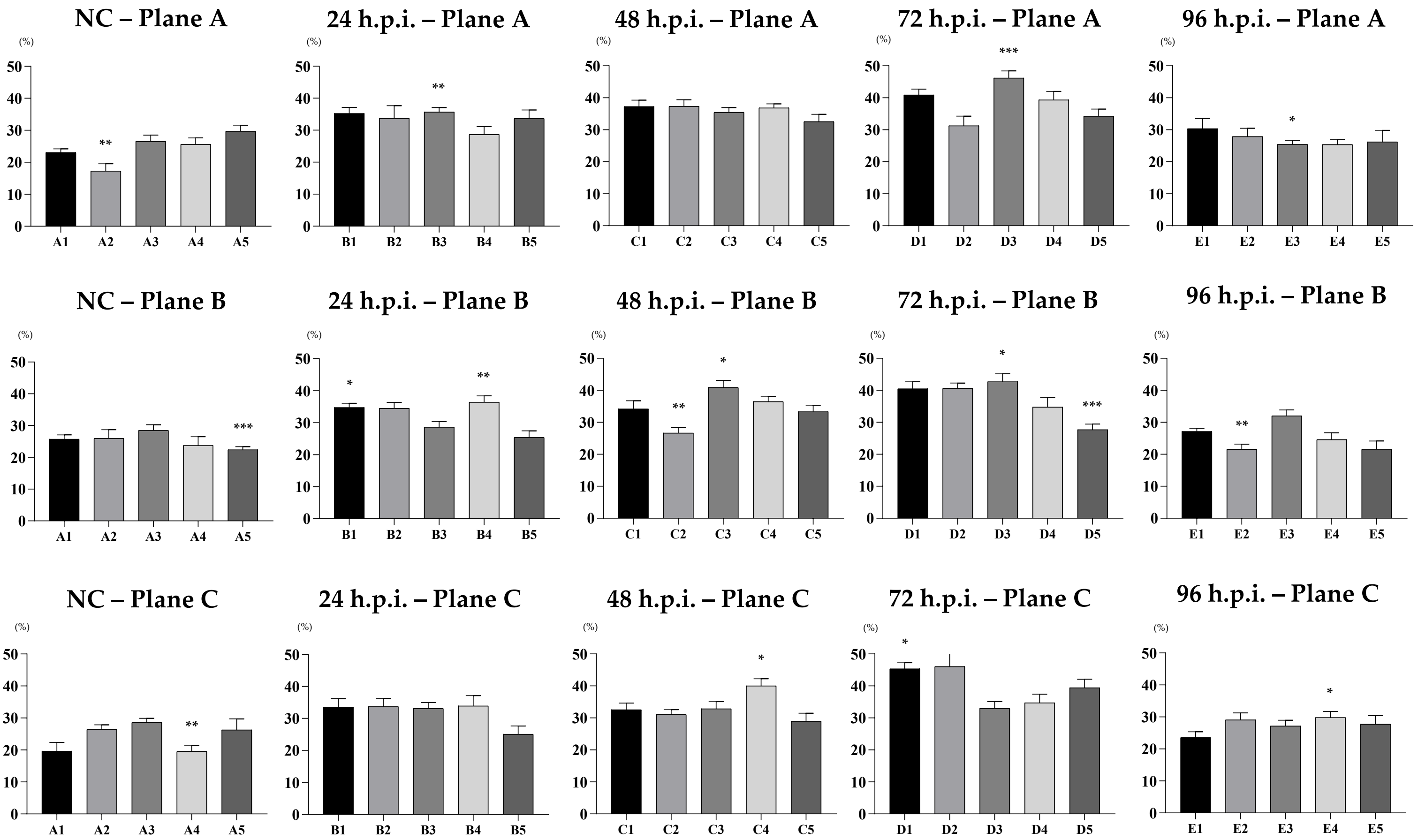


## Hepatocyte binucleation – Thirty images from three sectioning planes

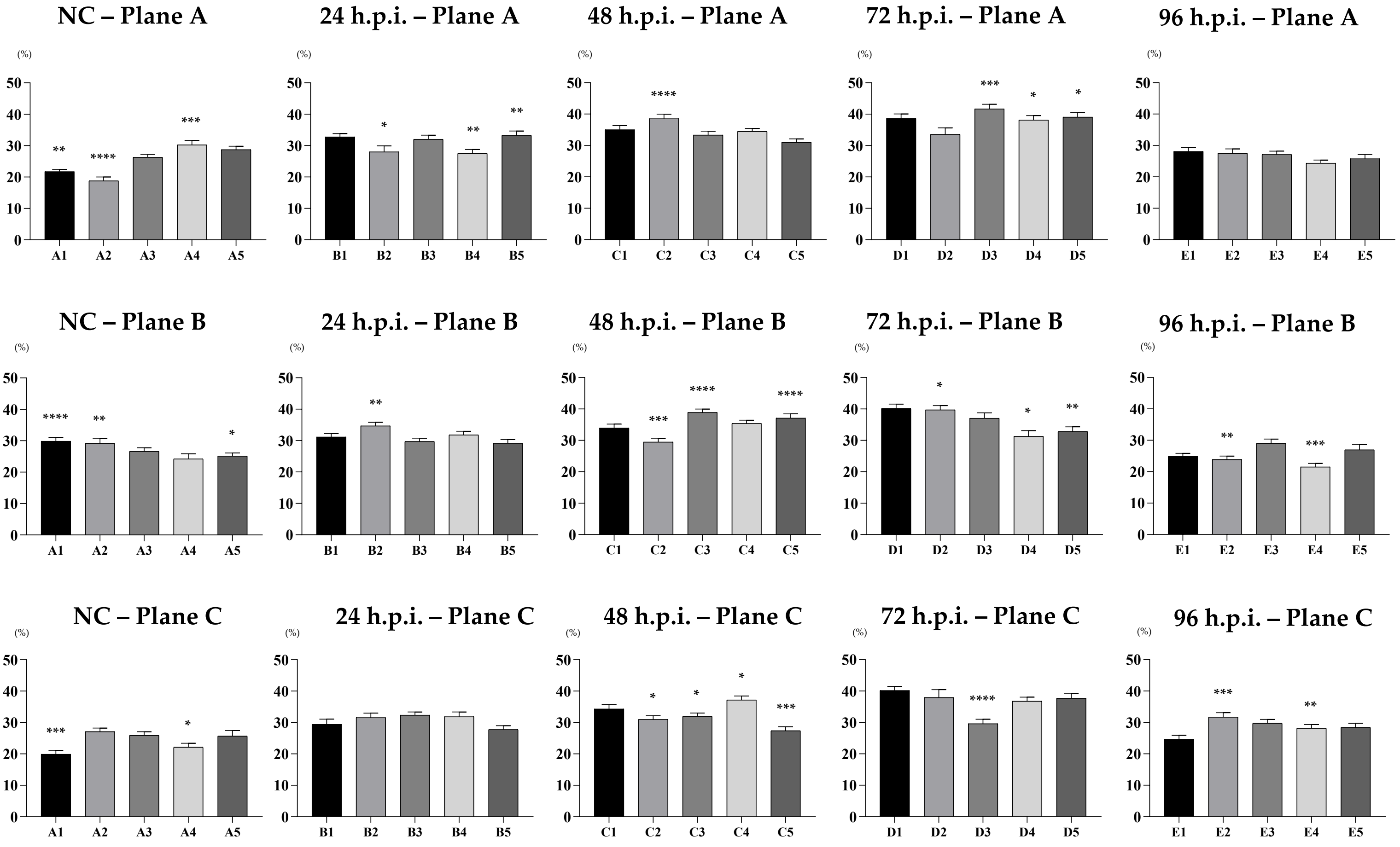


NC: Negative Control; h.p.i.: hours post-infection. Statistical analysis: unpaired t test with Welch's correction. *$p < 0.05$; **$p < 0.01$; ***$p < 0.001$; ****$p < 0.0001$.

# HEPATOCYTE BINUCLEATION – PART II

## Hepatocyte binucleation – Forty images from three sectioning planes

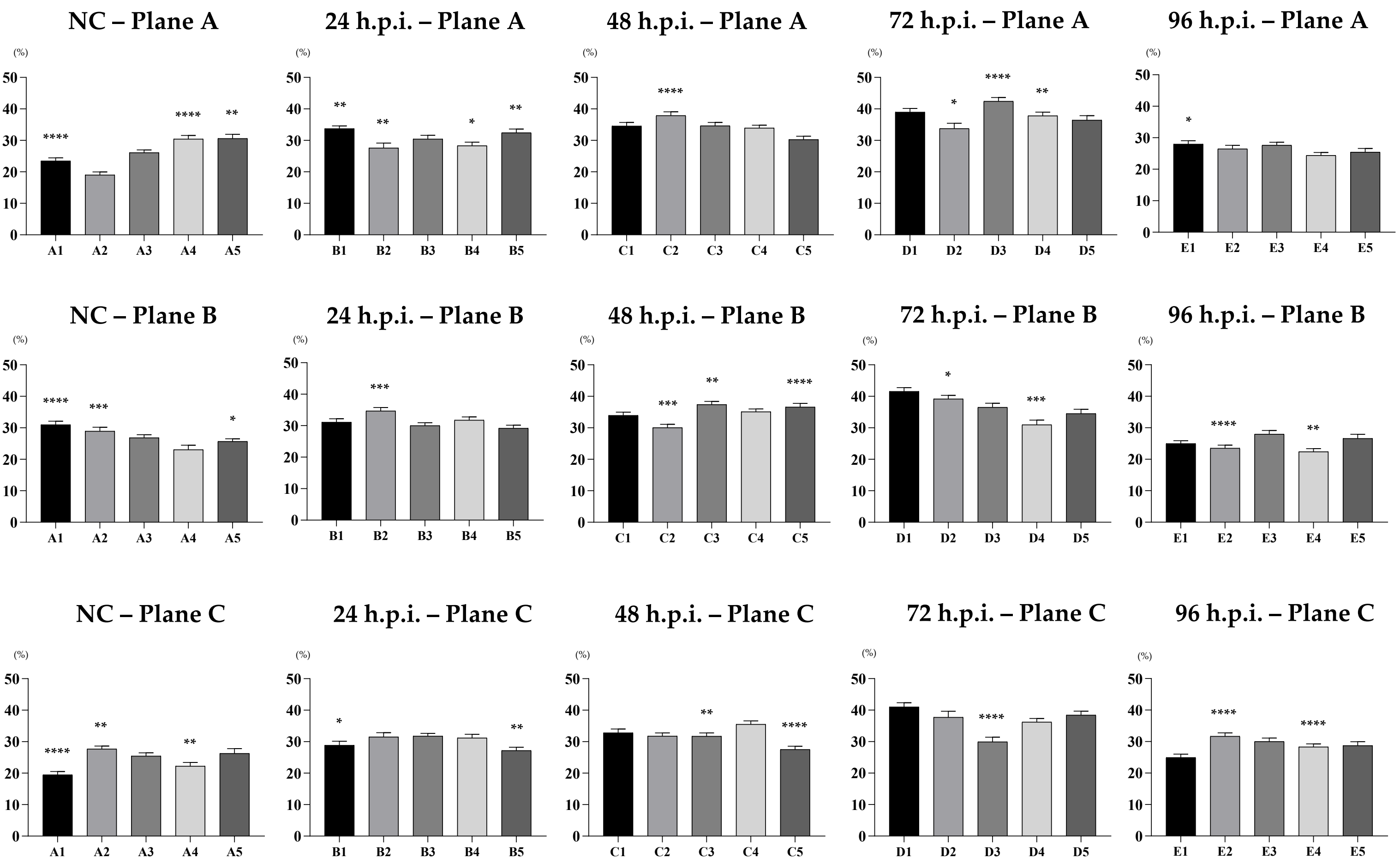


NC: Negative Control; h.p.i.: hours post-infection. Statistical analysis: unpaired t test with Welch's correction. *$p < 0.05$; **$p < 0.01$; ***$p < 0.001$; ****$p < 0.0001$.

# HEPATOCYTE BINUCLEATION – PART III

## Hepatocyte binucleation – Thirty images from three sectioning planes

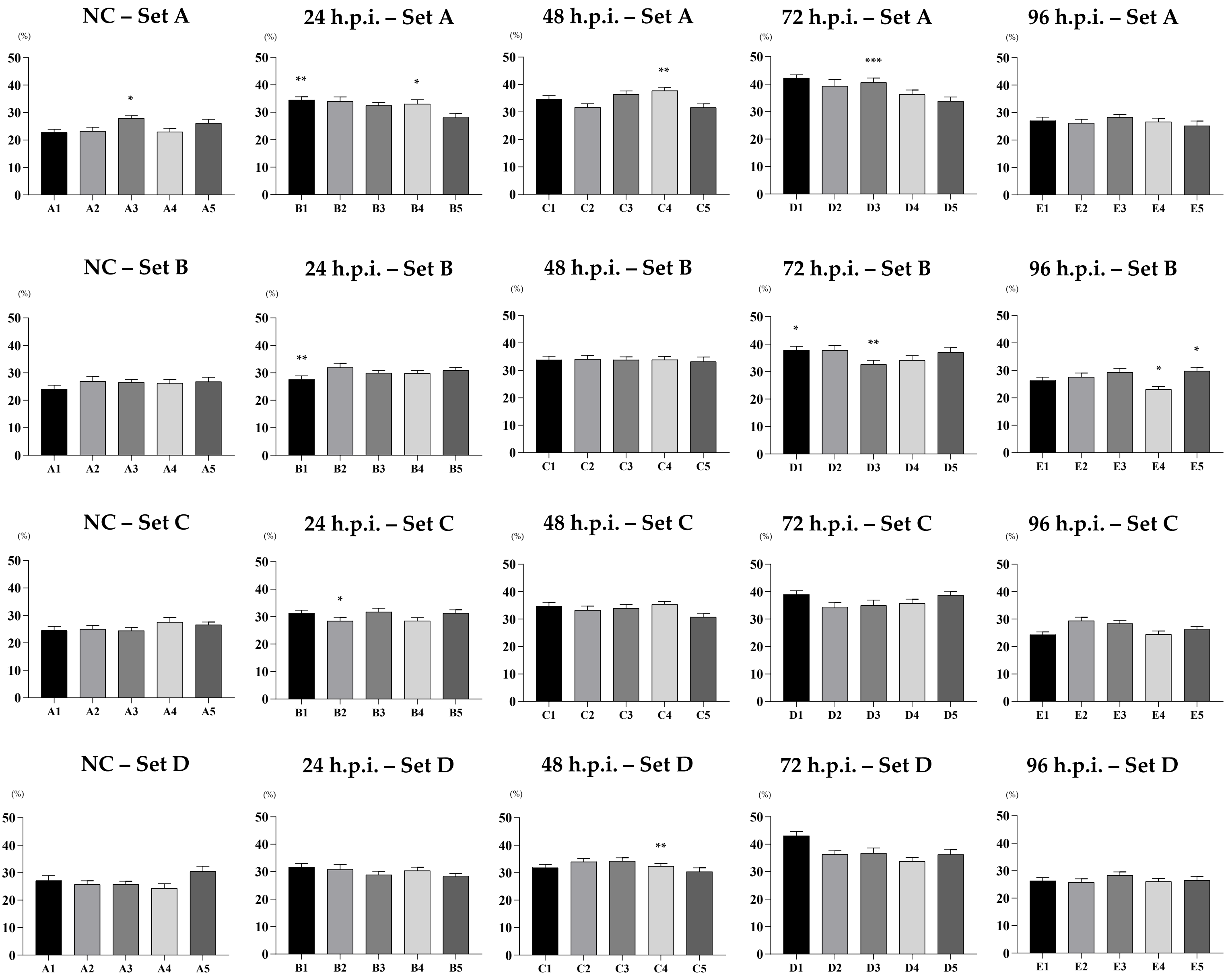


## Hepatocyte binucleation – Total (120 images)

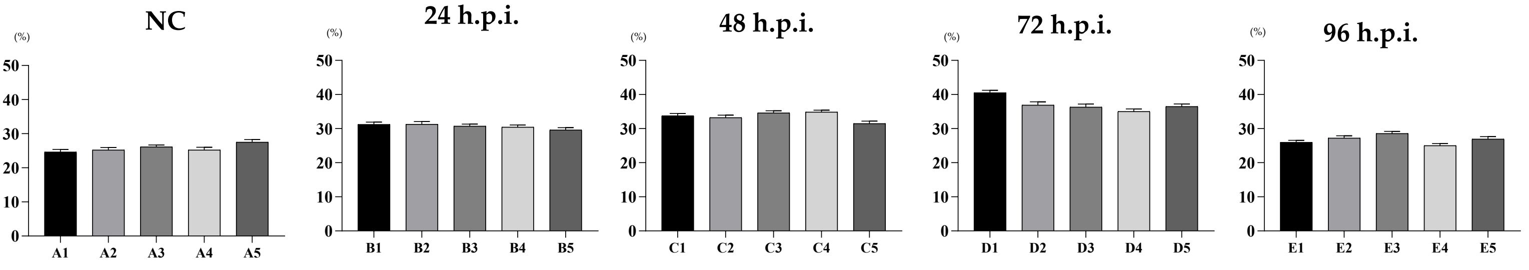


NC: Negative Control; h.p.i.: hours post-infection. Statistical analysis: unpaired t test with Welch's correction. *$p < 0.05$; **$p < 0.01$; ***$p < 0.001$; ****$p < 0.0001$.

# HEPATOCYTE BINUCLEATION – DESCRIPTIVE STATISTICS AND COMPARATIVE ANALYSIS NEGATIVE CONTROL – PART I

| | | | Animal 1 | Animal 2 | Animal 3 | Animal 4 | Animal 5 |
|---|---|---|---|---|---|---|---|
| Plane A | 10 Images | Subset Mean | 23.08 | 17.27 | 26.55 | 25.59 | 29.75 |
| | | (Total – Subset) Mean | 24.79 | 25.94 | 26.09 | 25.21 | 27.31 |
| | | p value | > 0.05 | 0.0039 | > 0.05 | > 0.05 | > 0.05 |
| | | Mean Difference ± SEM | 1.713 ± 1.371 | 8.667 ± 2.382 | -0.467 ± 2.005 | -0.383 ± 2.179 | -2.435 ± 2.011 |
| | 30 Images | Subset Mean | 21.71 | 18.82 | 26.30 | 30.27 | 28.72 |
| | | (Total – Subset) Mean | 25.62 | 27.35 | 26.07 | 23.57 | 27.11 |
| | | p value | 0.0014 | < 0.0001 | > 0.05 | 0.0001 | > 0.05 |
| | | Mean Difference ± SEM | 3.912 ± 1.192 | 8.527 ± 1.413 | -0.228 ± 1.182 | -6.701 ± 1.622 | -1.611 ± 1.430 |
| | 40 Images | Subset Mean | 23.48 | 19.01 | 26.09 | 30.43 | 30.59 |
| | | (Total – Subset) Mean | 25.23 | 28.32 | 26.14 | 22.65 | 25.98 |
| | | p value | > 0.05 | < 0.0001 | > 0.05 | < 0.0001 | 0.0053 |
| | | Mean Difference ± SEM | 1.751 ± 1.379 | 9.314 ± 1.243 | 0.052 ± 1.143 | -7.777 ± 1.462 | -4.612 ± 1.602 |
| Plane B | 10 Images | Subset Mean | 25.74 | 25.97 | 28.48 | 23.75 | 22.41 |
| | | (Total – Subset) Mean | 24.55 | 25.15 | 25.91 | 25.38 | 27.98 |
| | | p value | > 0.05 | > 0.05 | > 0.05 | > 0.05 | 0.0001 |
| | | Mean Difference ± SEM | -1.191 ± 1.550 | -0.818 ± 2.815 | -2.572 ± 1.891 | 1.628 ± 2.864 | 5.571 ± 1.244 |
| | 30 Images | Subset Mean | 29.84 | 29.15 | 26.60 | 24.24 | 25.15 |
| | | (Total – Subset) Mean | 22.91 | 23.91 | 25.97 | 25.58 | 28.30 |
| | | p value | < 0.0001 | 0.0035 | > 0.05 | > 0.05 | 0.0210 |
| | | Mean Difference ± SEM | -6.929 ± 1.472 | -5.240 ± 1.701 | -0.625 ± 1.331 | 1.335 ± 1.856 | 3.146 ± 1.339 |
| | 40 Images | Subset Mean | 31.00 | 28.96 | 26.85 | 23.08 | 25.67 |
| | | (Total – Subset) Mean | 21.47 | 23.35 | 25.77 | 26.32 | 28.43 |
| | | p value | < 0.0001 | 0.0003 | > 0.05 | > 0.05 | 0.0411 |
| | | Mean Difference ± SEM | -9.530 ± 1.322 | -5.607 ± 1.493 | -1.079 ± 1.179 | 3.249 ± 1.682 | 2.759 ± 1.335 |

SEM: Standard Error of the Mean.

**HEPATOCYTE BINUCLEATION – DESCRIPTIVE STATISTICS AND COMPARATIVE ANALYSIS NEGATIVE CONTROL – PART II**

| | | | Animal 1 | Animal 2 | Animal 3 | Animal 4 | Animal 5 |
|---|---|---|---|---|---|---|---|
| Plane C | 10 Images | Subset Mean | 19.64 | 26.45 | 28.66 | 19.56 | 26.29 |
| | | (Total – Subset) Mean | 25.10 | 25.11 | 25.90 | 25.76 | 27.62 |
| | | p value | > 0.05 | > 0.05 | > 0.05 | 0.0070 | > 0.05 |
| | | Mean Difference ± SEM | 5.466 ± 2.840 | -1.341 ± 1.593 | -2.763 ± 1.369 | 6.195 ± 1.947 | 1.337 ± 3.532 |
| | 30 Images | Subset Mean | 19.90 | 27.12 | 25.90 | 22.16 | 25.68 |
| | | (Total – Subset) Mean | 26.23 | 24.59 | 26.20 | 26.27 | 28.12 |
| | | p value | 0.0001 | > 0.05 | > 0.05 | 0.0107 | > 0.05 |
| | | Mean Difference ± SEM | 6.325 ± 1.513 | -2.529 ± 1.424 | 0.2982 ± 1.306 | 4.111 ± 1.565 | 2.446 ± 1.940 |
| | 40 Images | Subset Mean | 19.46 | 27.69 | 25.44 | 22.22 | 26.28 |
| | | (Total – Subset) Mean | 27.24 | 23.98 | 26.47 | 26.75 | 28.13 |
| | | p value | < 0.0001 | 0.0059 | > 0.05 | 0.0040 | > 0.05 |
| | | Mean Difference ± SEM | 7.779 ± 1.361 | -3.706 ± 1.319 | 1.027 ± 1.211 | 4.528 ± 1.533 | 1.854 ± 1.719 |
| 30 Images Multi-plane Sets | Set A | Subset Mean | 22.82 | 23.23 | 27.90 | 22.97 | 26.15 |
| | | (Total – Subset) Mean | 25.26 | 25.88 | 25.54 | 26.00 | 27.97 |
| | | p value | > 0.05 | > 0.05 | 0.0455 | > 0.05 | > 0.05 |
| | | Mean Difference ± SEM | 2.439 ± 1.456 | 2.651 ± 1.672 | -2.364 ± 1.157 | 3.031 ± 1.621 | 1.822 ± 1.663 |
| | Set B | Subset Mean | 24.11 | 26.90 | 26.46 | 26.11 | 26.80 |
| | | (Total – Subset) Mean | 24.82 | 24.66 | 26.01 | 24.95 | 27.75 |
| | | p value | > 0.05 | > 0.05 | > 0.05 | > 0.05 | > 0.05 |
| | | Mean Difference ± SEM | 0.715 ± 1.668 | -2.241 ± 1.864 | -0.447 ± 1.282 | -1.164 ± 1.765 | 0.949 ± 1.796 |
| | Set C | Subset Mean | 24.53 | 24.96 | 24.44 | 27.58 | 26.61 |
| | | (Total – Subset) Mean | 24.68 | 25.31 | 26.69 | 24.46 | 27.82 |
| | | p value | > 0.05 | > 0.05 | > 0.05 | > 0.05 | > 0.05 |
| | | Mean Difference ± SEM | 0.1537 ± 1.710 | 0.347 ± 1.595 | 2.255 ± 1.298 | -3.122 ± 1.910 | 1.209 ± 1.366 |
| | Set D | Subset Mean | 27.13 | 25.79 | 25.71 | 24.30 | 30.50 |
| | | (Total – Subset) Mean | 23.82 | 25.03 | 26.27 | 25.56 | 26.52 |
| | | p value | > 0.05 | > 0.05 | > 0.05 | > 0.05 | > 0.05 |
| | | Mean Difference ± SEM | -3.309 ± 1.931 | -0.757 ±1.548 | 0.556 ± 1.334 | 1.255 ± 1.917 | -3.981 ± 1.997 |

SEM: Standard Error of the Mean.

## HEPATOCYTE BINUCLEATION– DESCRIPTIVE STATISTICS AND COMPARATIVE ANALYSIS 24 HOURS POST-INFECTION – PART I

| | | | Animal 1 | Animal 2 | Animal 3 | Animal 4 | Animal 5 |
|---|---|---|---|---|---|---|---|
| Plane A | 10 Images | Subset Mean | 35.22 | 33.73 | 35.68 | 28.69 | 33.67 |
| | | (Total – Subset) Mean | 30.87 | 31.05 | 30.29 | 30.57 | 29.25 |
| | | p value | > 0.05 | > 0.05 | 0.0036 | > 0.05 | > 0.05 |
| | | Mean Difference ± SEM | -4.346 ± 2.001 | -2.684 ± 4.000 | -5.384 ± 1.506 | 1.885 ± 2.560 | -4.419 ± 2.718 |
| | 30 Images | Subset Mean | 32.77 | 28.02 | 32.03 | 27.57 | 33.26 |
| | | (Total – Subset) Mean | 30.72 | 32.36 | 30.31 | 31.37 | 28.40 |
| | | p value | > 0.05 | 0.0434 | > 0.05 | 0.0094 | 0.0030 |
| | | Mean Difference ± SEM | -2.054 ± 1.294 | 4.338 ± 2.082 | -1.718 ± 1.439 | 3.802 ± 1.410 | -4.853 ± 1.544 |
| | 40 Images | Subset Mean | 33.71 | 27.61 | 30.42 | 28.30 | 32.46 |
| | | (Total – Subset) Mean | 29.99 | 33.10 | 30.90 | 31.40 | 28.20 |
| | | p value | 0.0028 | 0.0030 | > 0.05 | 0.0221 | 0.0024 |
| | | Mean Difference ± SEM | -3.718 ± 1.213 | 5.489 ± 1.779 | 0.4780 ± 1.323 | 3.176 ± 1.359 | -4.261 ± 1.352 |
| Plane B | 10 Images | Subset Mean | 34.75 | 34.54 | 28.64 | 36.43 | 25.44 |
| | | (Total – Subset) Mean | 30.91 | 30.98 | 30.93 | 29.87 | 30.00 |
| | | p value | 0.0233 | > 0.05 | > 0.05 | 0.0086 | > 0.05 |
| | | Mean Difference ± SEM | -3.839 ± 1.511 | -3.563 ± 2.018 | 2.290 ± 1.799 | -6.558 ± 2.061 | 4.563 ± 2.143 |
| | 30 Images | Subset Mean | 31.14 | 34.71 | 29.71 | 31.83 | 29.20 |
| | | (Total – Subset) Mean | 31.26 | 30.13 | 31.09 | 29.95 | 29.76 |
| | | p value | > 0.05 | 0.0026 | > 0.05 | > 0.05 | > 0.05 |
| | | Mean Difference ± SEM | 0.120 ± 1.336 | -4.579 ± 1.471 | 1.374 ± 1.269 | -1.881 ± 1.386 | 0.552 ± 1.362 |
| | 40 Images | Subset Mean | 31.12 | 34.68 | 30.04 | 31.78 | 29.19 |
| | | (Total – Subset) Mean | 31.29 | 29.57 | 31.10 | 29.74 | 29.83 |
| | | p value | > 0.05 | 0.0010 | > 0.05 | > 0.05 | > 0.05 |
| | | Mean Difference ± SEM | 0.162 ± 1.335 | -5.119 ± 1.507 | 1.059 ± 1.161 | -2.041 ± 1.287 | 0.646 ± 1.267 |

SEM: Standard Error of the Mean.

## HEPATOCYTE BINUCLEATION– DESCRIPTIVE STATISTICS AND COMPARATIVE ANALYSIS 24 HOURS POST-INFECTION – PART II

| | | | Animal 1 | Animal 2 | Animal 3 | Animal 4 | Animal 5 |
|---|---|---|---|---|---|---|---|
| Plane C | 10 Images | Subset Mean | 33.53 | 33.68 | 33.09 | 33.89 | 25.08 |
| | | (Total – Subset) Mean | 31.02 | 31.05 | 30.53 | 30.10 | 30.03 |
| | | p value | > 0.05 | > 0.05 | > 0.05 | > 0.05 | > 0.05 |
| | | Mean Difference ± SEM | -2.507 ± 2.751 | -2.624 ± 2.729 | -2.561 ± 1.984 | -3.792 ± 3.257 | 4.954 ± 2.664 |
| | 30 Images | Subset Mean | 29.41 | 31.58 | 32.36 | 31.87 | 27.78 |
| | | (Total – Subset) Mean | 31.84 | 31.17 | 30.21 | 29.93 | 30.23 |
| | | p value | > 0.05 | > 0.05 | > 0.05 | > 0.05 | > 0.05 |
| | | Mean Difference ± SEM | 2.424 ± 1.762 | -0.405 ± 1.709 | -2.153 ± 1.218 | -1.931 ± 1.630 | 2.444 ± 1.404 |
| | 40 Images | Subset Mean | 28.86 | 31.52 | 31.77 | 31.17 | 27.21 |
| | | (Total – Subset) Mean | 32.42 | 31.15 | 30.23 | 30.04 | 30.82 |
| | | p value | 0.0179 | > 0.05 | > 0.05 | > 0.05 | 0.0065 |
| | | Mean Difference ± SEM | 3.556 ± 1.462 | -0.370 ± 1.680 | -1.537 ± 1.147 | -1.135 ± 1.417 | 3.614 ± 1.294 |
| 30 Images Multi-plane Sets | Set A | Subset Mean | 34.50 | 33.98 | 32.47 | 33.00 | 28.06 |
| | | (Total – Subset) Mean | 30.14 | 30.37 | 30.17 | 29.55 | 30.14 |
| | | p value | 0.0023 | > 0.05 | > 0.05 | 0.0490 | > 0.05 |
| | | Mean Difference ± SEM | -4.356 ± 1.364 | -3.614 ± 1.865 | -2.304 ± 1.264 | -3.449 ± 1.699 | 2.077 ± 1.684 |
| | Set B | Subset Mean | 27.62 | 31.93 | 29.92 | 29.82 | 30.90 |
| | | (Total – Subset) Mean | 32.44 | 31.05 | 31.02 | 30.62 | 29.19 |
| | | p value | 0.0020 | > 0.05 | > 0.05 | > 0.05 | > 0.05 |
| | | Mean Difference ± SEM | 4.819 ± 1.471 | -0.878 ± 1.828 | 1.097 ± 1.192 | 0.800 ± 1.339 | -1.711 ± 1.329 |
| | Set C | Subset Mean | 31.21 | 28.39 | 31.71 | 28.44 | 31.28 |
| | | (Total – Subset) Mean | 31.24 | 32.23 | 30.42 | 31.08 | 29.06 |
| | | p value | > 0.05 | 0.0258 | > 0.05 | > 0.05 | > 0.05 |
| | | Mean Difference ± SEM | 0.026 ± 1.376 | 3.845 ± 1.682 | -1.290 ± 1.474 | 2.639 ± 1.390 | -2.222 ± 1.402 |
| | Set D | Subset Mean | 31.60 | 30.79 | 28.87 | 30.41 | 28.23 |
| | | (Total – Subset) Mean | 31.11 | 31.43 | 31.37 | 30.42 | 30.08 |
| | | p value | > 0.05 | > 0.05 | > 0.05 | > 0.05 | > 0.05 |
| | | Mean Difference ± SEM | -0.490 ± 1.533 | 0.646 ± 2.015 | 2.497 ± 1.273 | 0.010 ± 1.445 | 1.856 ± 1.377 |

SEM: Standard Error of the Mean.

# HEPATOCYTE BINUCLEATION– DESCRIPTIVE STATISTICS AND COMPARATIVE ANALYSIS 48 HOURS POST-INFECTION– PART I

| | | | Animal 1 | Animal 2 | Animal 3 | Animal 4 | Animal 5 |
|---|---|---|---|---|---|---|---|
| Plane A | 10 Images | Subset Mean | 37.27 | 37.36 | 35.48 | 36.85 | 32.58 |
| | | (Total – Subset) Mean | 33.46 | 32.87 | 34.51 | 34.67 | 31.36 |
| | | p value | > 0.05 | > 0.05 | > 0.05 | > 0.05 | > 0.05 |
| | | Mean Difference ± SEM | -3.813 ± 2.098 | -4.496 ± 2.169 | -0.969 ± 1.597 | -2.178 ± 1.403 | -1.219 ± 2.408 |
| | 30 Images | Subset Mean | 35.03 | 38.54 | 33.33 | 34.47 | 31.02 |
| | | (Total – Subset) Mean | 33.36 | 31.48 | 35.01 | 34.98 | 31.61 |
| | | p value | > 0.05 | < 0.0001 | > 0.05 | > 0.05 | > 0.05 |
| | | Mean Difference ± SEM | -1.671 ± 1.532 | -7.064 ± 1.587 | 1.681 ± 1.426 | 0.504 ± 1.153 | 0.591 ± 1.389 |
| | 40 Images | Subset Mean | 23.48 | 19.01 | 26.09 | 30.43 | 30.59 |
| | | (Total – Subset) Mean | 25.23 | 28.32 | 26.14 | 22.65 | 25.98 |
| | | p value | > 0.05 | < 0.0001 | > 0.05 | < 0.0001 | 0.0053 |
| | | Mean Difference ± SEM | 1.751 ± 1.379 | 9.314 ± 1.243 | 0.052 ± 1.143 | -7.777 ± 1.462 | -4.612 ± 1.602 |
| Plane B | 10 Images | Subset Mean | 34.17 | 26.64 | 40.89 | 36.46 | 33.32 |
| | | (Total – Subset) Mean | 33.74 | 33.84 | 34.02 | 34.70 | 31.30 |
| | | p value | > 0.05 | 0.0023 | 0.0115 | > 0.05 | > 0.05 |
| | | Mean Difference ± SEM | -0.436 ± 2.626 | 7.202 ± 1.876 | -6.875 ± 2.252 | -1.762 ± 1.764 | -2.028 ± 2.180 |
| | 30 Images | Subset Mean | 33.94 | 29.47 | 38.91 | 35.39 | 37.09 |
| | | (Total – Subset) Mean | 33.72 | 34.50 | 33.15 | 34.67 | 29.59 |
| | | p value | > 0.05 | 0.0004 | < 0.0001 | > 0.05 | < 0.0001 |
| | | Mean Difference ± SEM | -0.218 ± 1.470 | 5.030 ± 1.336 | -5.764 ± 1.289 | -0.718 ± 1.240 | -7.495 ± 1.533 |
| | 40 Images | Subset Mean | 31.00 | 28.96 | 26.85 | 23.08 | 25.67 |
| | | (Total – Subset) Mean | 21.47 | 23.35 | 25.77 | 26.32 | 28.43 |
| | | p value | < 0.0001 | 0.003 | > 0.05 | > 0.05 | 0.0411 |
| | | Mean Difference ± SEM | -9.530 ± 1.322 | -5.607 ± 1.493 | -1.079 ± 1.179 | 3.249 ± 1.682 | 2.759 ± 1.335 |

SEM: Standard Error of the Mean.

## HEPATOCYTE BINUCLEATION – DESCRIPTIVE STATISTICS AND COMPARATIVE ANALYSIS 48 HOURS POST-INFECTION – PART II

| | | | Animal 1 | Animal 2 | Animal 3 | Animal 4 | Animal 5 |
|---|---|---|---|---|---|---|---|
| Plane C | 10 Images | Subset Mean | 32.54 | 31.09 | 32.84 | 40.00 | 28.99 |
| | | (Total – Subset) Mean | 33.89 | 33.44 | 34.75 | 34.38 | 31.69 |
| | | p value | > 0.05 | > 0.05 | > 0.05 | 0.0327 | > 0.05 |
| | | Mean Difference ± SEM | 1.343 ± 2.213 | 2.348 ± 1.641 | 1.910 ± 2.312 | -5.622 ± 2.274 | 2.698 ± 2.608 |
| | 30 Images | Subset Mean | 34.30 | 30.97 | 31.88 | 37.17 | 27.41 |
| | | (Total – Subset) Mean | 33.60 | 34.00 | 35.49 | 34.07 | 32.82 |
| | | p value | > 0.05 | 0.0366 | 0.0103 | 0.0328 | 0.0006 |
| | | Mean Difference ± SEM | -0.703 ± 1.570 | 3.036 ± 1.420 | 3.609 ± 1356 | -3.098 ± 1.403 | 5.407 ± 1.480 |
| | 40 Images | Subset Mean | 19.46 | 27.69 | 25.44 | 22.22 | 26.28 |
| | | (Total – Subset) Mean | 27.24 | 23.98 | 26.47 | 26.75 | 28.13 |
| | | p value | < 0.0001 | 0.0059 | > 0.05 | 0.0040 | > 0.05 |
| | | Mean Difference ± SEM | 7.779 ± 1.361 | -3.706 ± 1.319 | 1.027 ± 1.211 | 4.528 ± 1.533 | 1.854 ± 1.719 |
| 30 Images Multi-plane Sets | Set A | Subset Mean | 34.66 | 31.70 | 36.40 | 37.77 | 31.63 |
| | | (Total – Subset) Mean | 33.48 | 33.76 | 33.98 | 33.88 | 31.41 |
| | | p value | > 0.05 | > 0.05 | > 0.05 | 0.0020 | > 0.05 |
| | | Mean Difference ± SEM | -1.184 ± 1.494 | 2.059 ± 1.510 | -2.418 ± 1.452 | -3.896 ± 1.195 | -0.223 ± 1.566 |
| | Set B | Subset Mean | 33.81 | 34.05 | 33.81 | 33.86 | 33.17 |
| | | (Total – Subset) Mean | 33.76 | 32.97 | 34.85 | 35.18 | 30.90 |
| | | p value | > 0.05 | > 0.05 | > 0.05 | > 0.05 | > 0.05 |
| | | Mean Difference ± SEM | -0.051 ± 1.562 | -1.081 ± 1.620 | 1.034 ± 1.348 | 1.321 ± 1.333 | -2.272 ± 1.834 |
| | Set C | Subset Mean | 34.79 | 33.22 | 33.91 | 35.40 | 30.72 |
| | | (Total – Subset) Mean | 33.43 | 33.25 | 34.82 | 34.66 | 31.72 |
| | | p value | > 0.05 | > 0.05 | > 0.05 | > 0.05 | > 0.05 |
| | | Mean Difference ± SEM | -1.357 ± 1.517 | 0.024 ± 1.733 | 0.910 ± 1.562 | -0.738 ± 1.219 | 0.999 ± 1.501 |
| | Set D | Subset Mean | 31.83 | 33.99 | 34.23 | 32.37 | 30.34 |
| | | (Total – Subset) Mean | 34.42 | 32.99 | 34.71 | 35.68 | 31.84 |
| | | p value | > 0.05 | > 0.05 | > 0.05 | 0.0055 | > 0.05 |
| | | Mean Difference ± SEM | 2.592 ± 1.412 | -1.002 ± 1.462 | 0.474 ± 1.428 | 3.312 ± 1.148 | 1.497 ± 1.638 |

SEM: Standard Error of the Mean.

## HEPATOCYTE BINUCLEATION– DESCRIPTIVE STATISTICS AND COMPARATIVE ANALYSIS 72 HOURS POST-INFECTION– PART I

| | | | Animal 1 | Animal 2 | Animal 3 | Animal 4 | Animal 5 |
|---|---|---|---|---|---|---|---|
| Plane A | 10 Images | Subset Mean | 40.91 | 31.29 | 46.17 | 39.35 | 34.28 |
| | | (Total – Subset) Mean | 40.49 | 37.41 | 35.40 | 34.61 | 36.66 |
| | | p value | > 0.05 | > 0.05 | 0.0007 | > 0.05 | > 0.05 |
| | | Mean Difference ± SEM | -0.429 ± 1.980 | 6.118 ± 3.129 | -10.77 ± 2.410 | -4.738 ± 2.801 | 2.376 ± 2.339 |
| | 30 Images | Subset Mean | 38.67 | 33.58 | 41.69 | 38.13 | 39.05 |
| | | (Total – Subset) Mean | 41.14 | 38.01 | 34.49 | 33.96 | 35.60 |
| | | p value | > 0.05 | > 0.05 | 0.0001 | 0.0147 | 0.0495 |
| | | Mean Difference ± SEM | 2.469 ± 1.623 | 4.428 ± 2.316 | -7.201 ± 1.775 | -4.171 ± 1.655 | -3.455 ± 1.720 |
| | 40 Images | Subset Mean | 39.00 | 33.77 | 42.42 | 37.80 | 36.43 |
| | | (Total – Subset) Mean | 41.28 | 38.46 | 33.23 | 33.60 | 36.47 |
| | | p value | > 0.05 | 0.0210 | < 0.0001 | 0.0070 | >0.05 |
| | | Mean Difference ± SEM | 2.285 ± 1.465 | 4.699 ± 1.993 | -9.194 ± 1.577 | -4.198 ± 1.521 | 0.048 ± 1.713 |
| Plane B | 10 Images | Subset Mean | 40.46 | 40.60 | 42.71 | 34.78 | 27.71 |
| | | (Total – Subset) Mean | 40.53 | 36.56 | 35.71 | 35.02 | 37.25 |
| | | p value | > 0.05 | > 0.05 | > 0.05 | > 0.05 | 0.0002 |
| | | Mean Difference ± SEM | 0.063 ± 2.299 | -4.034 ± 1.967 | -6.996 ± 2.597 | 0.245 ± 3.121 | 9.550 ± 1.912 |
| | 30 Images | Subset Mean | 40.19 | 39.75 | 37.08 | 31.28 | 32.81 |
| | | (Total – Subset) Mean | 40.63 | 35.95 | 36.03 | 36.24 | 37.68 |
| | | p value | > 0.05 | 0.0351 | > 0.05 | 0.0168 | 0.0074 |
| | | Mean Difference ± SEM | 0.446 ± 1.619 | -3.799 ± 1.770 | -1.048 ± 1.975 | 4.960 ± 1.989 | 4.869 ± 1.745 |
| | 40 Images | Subset Mean | 41.56 | 39.21 | 36.51 | 30.98 | 34.50 |
| | | (Total – Subset) Mean | 40.00 | 35.74 | 36.19 | 37.01 | 37.44 |
| | | p value | > 0.05 | 0.0446 | > 0.05 | 0.0006 | > 0.05 |
| | | Mean Difference ± SEM | -1.564 ± 1.508 | -3.463 ± 1.705 | -0.324 ± 1.747 | 6.038 ± 1.668 | 2.932 ± 1.692 |

SEM: Standard Error of the Mean.

**HEPATOCYTE BINUCLEATION– DESCRIPTIVE STATISTICS AND COMPARATIVE ANALYSIS 72 HOURS POST-INFECTION– PART II**

| | | | Animal 1 | Animal 2 | Animal 3 | Animal 4 | Animal 5 |
|---|---|---|---|---|---|---|---|
| Plane C | 10 Images | Subset Mean | 45.37 | 46.07 | 33.04 | 34.73 | 39.44 |
| | | (Total – Subset) Mean | 40.08 | 36.06 | 36.59 | 35.03 | 36.19 |
| | | p value | 0.0226 | > 0.05 | > 0.05 | > 0.05 | > 0.05 |
| | | Mean Difference ± SEM | -5.288 ± 2.024 | -10.01 ± 5.528 | 3.548 ± 2.306 | 0.299 ± 2.824 | -3.247 ± 2.809 |
| | 30 Images | Subset Mean | 40.17 | 37.94 | 29.61 | 36.76 | 37.71 |
| | | (Total – Subset) Mean | 40.64 | 36.55 | 38.52 | 34.41 | 36.04 |
| | | p value | > 0.05 | > 0.05 | < 0.0001 | > 0.05 | > 0.05 |
| | | Mean Difference ± SEM | 0.471 ± 1.577 | -1.394 ± 2.664 | 8.907 ± 1.707 | -2.350 ± 1.588 | -1.667 ± 1.709 |
| | 40 Images | Subset Mean | 41.00 | 37.72 | 29.95 | 36.23 | 38.45 |
| | | (Total – Subset) Mean | 40.28 | 36.49 | 39.47 | 34.39 | 35.47 |
| | | p value | > 0.05 | > 0.05 | < 0.0001 | > 0.05 | > 0.05 |
| | | Mean Difference ± SEM | - 0.721 ± 1.598 | -1.236 ± 2.194 | 9.518 ± 1.719 | -1.840 ± 1.518 | -2.980 ± 1.586 |
| 30 Images Multi-plane Sets | Set A | Subset Mean | 42.25 | 39.32 | 40.64 | 36.28 | 33.81 |
| | | (Total – Subset) Mean | 39.95 | 36.09 | 34.84 | 34.57 | 37.34 |
| | | p value | > 0.05 | > 0.05 | 0.0038 | > 0.05 | > 0.05 |
| | | Mean Difference ± SEM | -2.303 ± 1.458 | -3.228 ± 2.554 | -5.794 ± 1.915 | -1.709 ± 1.837 | 3.536 ± 1.780 |
| | Set B | Subset Mean | 37.78 | 37.78 | 32.71 | 34.13 | 37.03 |
| | | (Total – Subset) Mean | 41.43 | 36.60 | 37.49 | 35.29 | 36.27 |
| | | p value | 0.0334 | > 0.05 | 0.0098 | > 0.05 | > 0.05 |
| | | Mean Difference ± SEM | 3.649 ± 1.666 | -1.180 ± 2.100 | 4.782 ± 1.794 | 1.167 ± 1.898 | -0.760 ± 1.896 |
| | Set C | Subset Mean | 38.99 | 34.16 | 35.04 | 35.77 | 38.73 |
| | | (Total – Subset) Mean | 41.03 | 37.58 | 36.43 | 34.26 | 35.90 |
| | | p value | > 0.05 | > 0.05 | > 0.05 | > 0.05 | > 0.05 |
| | | Mean Difference ± SEM | 2.039 ± 1.555 | 3.417 ± 2.176 | 1.389 ± 2.109 | -1.505 ± 1.743 | -2.285 ± 1.536 |
| | Set D | Subset Mean | 43.06 | 36.33 | 36.79 | 33.83 | 36.27 |
| | | (Total – Subset) Mean | 39.67 | 37.09 | 36.13 | 35.39 | 36.52 |
| | | p value | > 0.05 | > 0.05 | > 0.05 | > 0.05 | > 0.05 |
| | | Mean Difference ± SEM | -3.385 ± 1.779 | 0.764 ± 1.733 | -0.658 ± 2.111 | 1.561 ± 1.652 | 0.252 ± 1.972 |

SEM: Standard Error of the Mean.

**HEPATOCYTE BINUCLEATION– DESCRIPTIVE STATISTICS AND COMPARATIVE ANALYSIS 96 HOURS POST-INFECTION – PART I**

| | | | Animal 1 | Animal 2 | Animal 3 | Animal 4 | Animal 5 |
|---|---|---|---|---|---|---|---|
| Plane A | 10 Images | Subset Mean | 30.35 | 27.91 | 25.44 | 25.38 | 26.22 |
| | | (Total – Subset) Mean | 25.58 | 27.16 | 28.83 | 25.00 | 26.98 |
| | | p value | > 0.05 | > 0.05 | 0.0335 | > 0.05 | > 0.05 |
| | | Mean Difference ± SEM | -4.772 ± 3.266 | -0.752 ± 2.681 | 3.393 ± 1.442 | -0.387 ± 1.640 | 0.758 ± 3.693 |
| | 30 Images | Subset Mean | 28.10 | 27.49 | 27.12 | 24.33 | 25.77 |
| | | (Total – Subset) Mean | 25.27 | 27.13 | 29.03 | 25.26 | 27.30 |
| | | p value | > 0.05 | > 0.05 | > 0.05 | > 0.05 | > 0.05 |
| | | Mean Difference ± SEM | -2.838 ± 1.431 | -0.359 ± 1.597 | 1.908 ± 1.290 | 0.928 ± 1.231 | 1.525 ± 1.662 |
| | 40 Images | Subset Mean | 27.99 | 26.48 | 27.64 | 24.41 | 25.42 |
| | | (Total – Subset) Mean | 24.97 | 27.59 | 29.01 | 25.34 | 27.67 |
| | | p value | 0.0204 | > 0.05 | > 0.05 | > 0.05 | > 0.05 |
| | | Mean Difference ± SEM | -3.024 ± 1.275 | 1.117 ± 1.433 | 1.373 ± 1.234 | 0.934 ± 1.179 | 2.245 ± 1.494 |
| Plane B | 10 Images | Subset Mean | 27.14 | 21.57 | 32.02 | 24.62 | 21.60 |
| | | (Total – Subset) Mean | 25.87 | 27.74 | 28.24 | 25.07 | 27.40 |
| | | p value | > 0.05 | 0.0039 | > 0.05 | > 0.05 | > 0.05 |
| | | Mean Difference ± SEM | -1.273 ± 1.199 | 6.163 ± 1.757 | -3.782 ± 1.935 | 0.451 ± 2.211 | 5.799 ± 2.650 |
| | 30 Images | Subset Mean | 24.87 | 23.91 | 29.00 | 21.53 | 26.97 |
| | | (Total – Subset) Mean | 26.34 | 28.32 | 28.40 | 26.20 | 26.90 |
| | | p value | > 0.05 | 0.0015 | > 0.05 | 0.0008 | > 0.05 |
| | | Mean Difference ± SEM | 1.476 ± 1.219 | 4.412 ± 1.332 | -0.599 ± 1.566 | 4.668 ± 1.305 | -0.065 ± 1.836 |
| | 40 Images | Subset Mean | 25.00 | 23.51 | 27.98 | 22.38 | 26.62 |
| | | (Total – Subset) Mean | 26.46 | 29.08 | 28.84 | 26.36 | 27.07 |
| | | p value | > 0.05 | < 0.0001 | > 0.05 | 0.0015 | > 0.05 |
| | | Mean Difference ± SEM | 1.467 ± 1.178 | 5.574 ± 1.287 | 0.863 ± 1.387 | 3.977 ± 1.205 | 0.449 ± 1.587 |

SEM: Standard Error of the Mean.

## HEPATOCYTE BINUCLEATION– DESCRIPTIVE STATISTICS AND COMPARATIVE ANALYSIS 96 HOURS POST-INFECTION – PART II

| | | | Animal 1 | Animal 2 | Animal 3 | Animal 4 | Animal 5 |
|---|---|---|---|---|---|---|---|
| Plane C | 10 Images | Subset Mean | 23.57 | 29.09 | 27.17 | 29.79 | 27.79 |
| | | (Total – Subset) Mean | 26.19 | 27.05 | 28.68 | 24.60 | 26.84 |
| | | p value | > 0.05 | > 0.05 | > 0.05 | 0.0247 | > 0.05 |
| | | Mean Difference ± SEM | 2.620 ± 1.897 | -2.043 ± 2.306 | 1.502 ± 1.907 | -5.198 ± 1.996 | -0.948 ± 2.749 |
| | 30 Images | Subset Mean | 24.63 | 31.75 | 29.77 | 28.20 | 28.40 |
| | | (Total – Subset) Mean | 26.42 | 25.71 | 28.14 | 23.97 | 26.42 |
| | | p value | > 0.05 | 0.0003 | > 0.05 | 0.0023 | > 0.05 |
| | | Mean Difference ± SEM | 1.798 ± 1.451 | -6.032 ± 1.547 | -1.629 ± 1.395 | -4.227 ± 1.317 | -1.973 ± 1.568 |
| | 40 Images | Subset Mean | 24.94 | 31.68 | 30.04 | 28.30 | 28.71 |
| | | (Total – Subset) Mean | 26.94 | 24.99 | 27.81 | 23.39 | 26.02 |
| | | p value | > 0.05 | < 0.0001 | > 0.05 | < 0.0001 | > 0.05 |
| | | Mean Difference ± SEM | 1.557 ± 1.303 | -6.691 ± 1.327 | -2.236 ± 1.293 | -4.912 ± 1.178 | -2.694 ± 1.503 |
| 30 Images Multi-plane Sets | Set A | Subset Mean | 27.02 | 26.19 | 28.21 | 26.60 | 25.20 |
| | | (Total – Subset) Mean | 25.63 | 27.56 | 28.66 | 24.51 | 27.49 |
| | | p value | > 0.05 | > 0.05 | > 0.05 | > 0.05 | > 0.05 |
| | | Mean Difference ± SEM | -1.396 ± 1.486 | 1.372 ± 1.571 | 0.453 ± 1.293 | -2.091 ± 1.315 | 2.285 ± 1.893 |
| | Set B | Subset Mean | 26.25 | 27.57 | 29.32 | 23.05 | 29.76 |
| | | (Total – Subset) Mean | 25.88 | 27.11 | 28.29 | 25.69 | 25.97 |
| | | p value | > 0.05 | > 0.05 | > 0.05 | 0.0486 | 0.0211 |
| | | Mean Difference ± SEM | -0.368 ± 1.428 | -0.464 ± 1.697 | -1.026 ± 1.585 | 2.645 ± 1.310 | -3.792 ± 1.595 |
| | Set C | Subset Mean | 24.33 | 29.39 | 28.36 | 24.42 | 26.17 |
| | | (Total – Subset) Mean | 26.53 | 26.50 | 28.61 | 25.23 | 27.17 |
| | | p value | > 0.05 | > 0.05 | > 0.05 | > 0.05 | > 0.05 |
| | | Mean Difference ± SEM | 2.200 ± 1.233 | -2.888 ± 1.513 | 0.2537 ± 1.409 | 0.816 ± 1.442 | 0.993 ± 1.475 |
| | Set D | Subset Mean | 26.30 | 25.74 | 28.31 | 26.06 | 26.53 |
| | | (Total – Subset) Mean | 25.87 | 27.72 | 28.63 | 24.69 | 27.05 |
| | | p value | > 0.05 | > 0.05 | > 0.05 | > 0.05 | > 0.05 |
| | | Mean Difference ± SEM | -0.436 ± 1.365 | 1.979 ± 1.534 | 0.319 ± 1.452 | -1.370 ± 1.326 | 0.514 ± 1.640 |

SEM: Standard Error of the Mean.

# SINUSOIDAL DILATION – PART I

## Sinusoidal dilation – Ten images from one sectioning plane

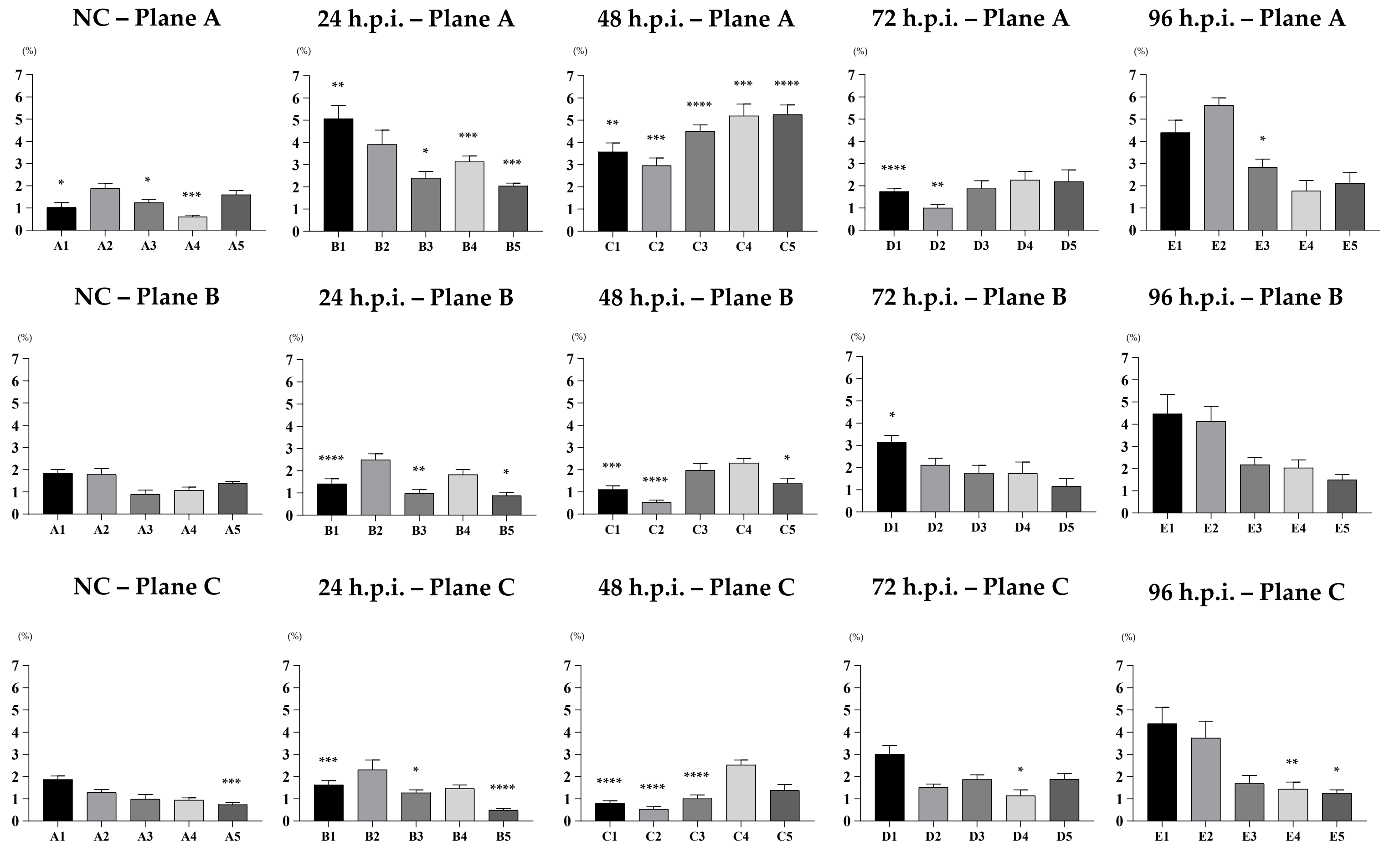


## Sinusoidal dilation – Thirty images from one sectioning plane

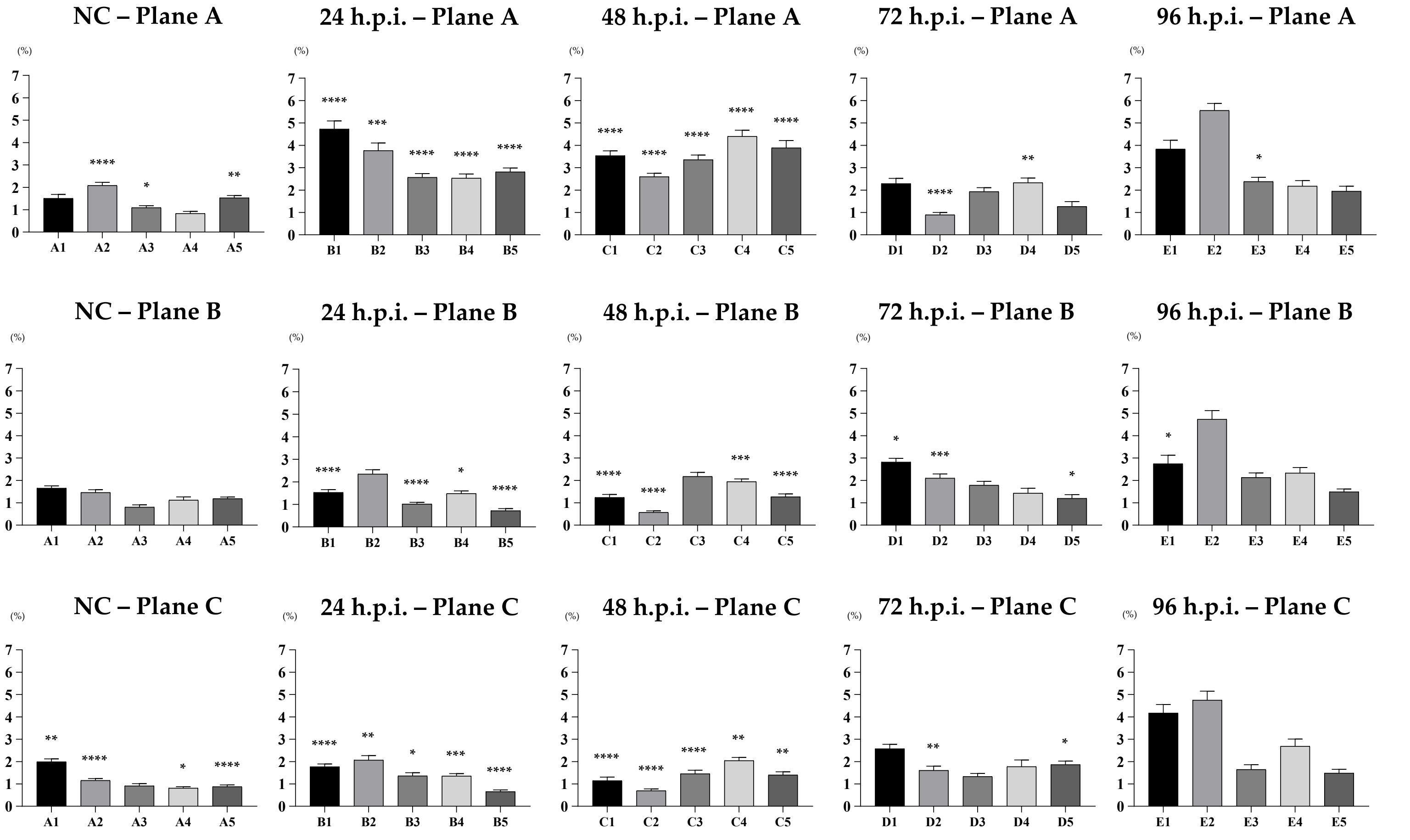


NC: Negative Control; h.p.i.: hours post-infection. Statistical analysis: unpaired t test with Welch's correction. *$p < 0.05$; **$p < 0.01$; ***$p < 0.001$; ****$p < 0.0001$.

# SINUSOIDAL DILATION – PART II

## Sinusoidal dilation – Forty images from one sectioning plane

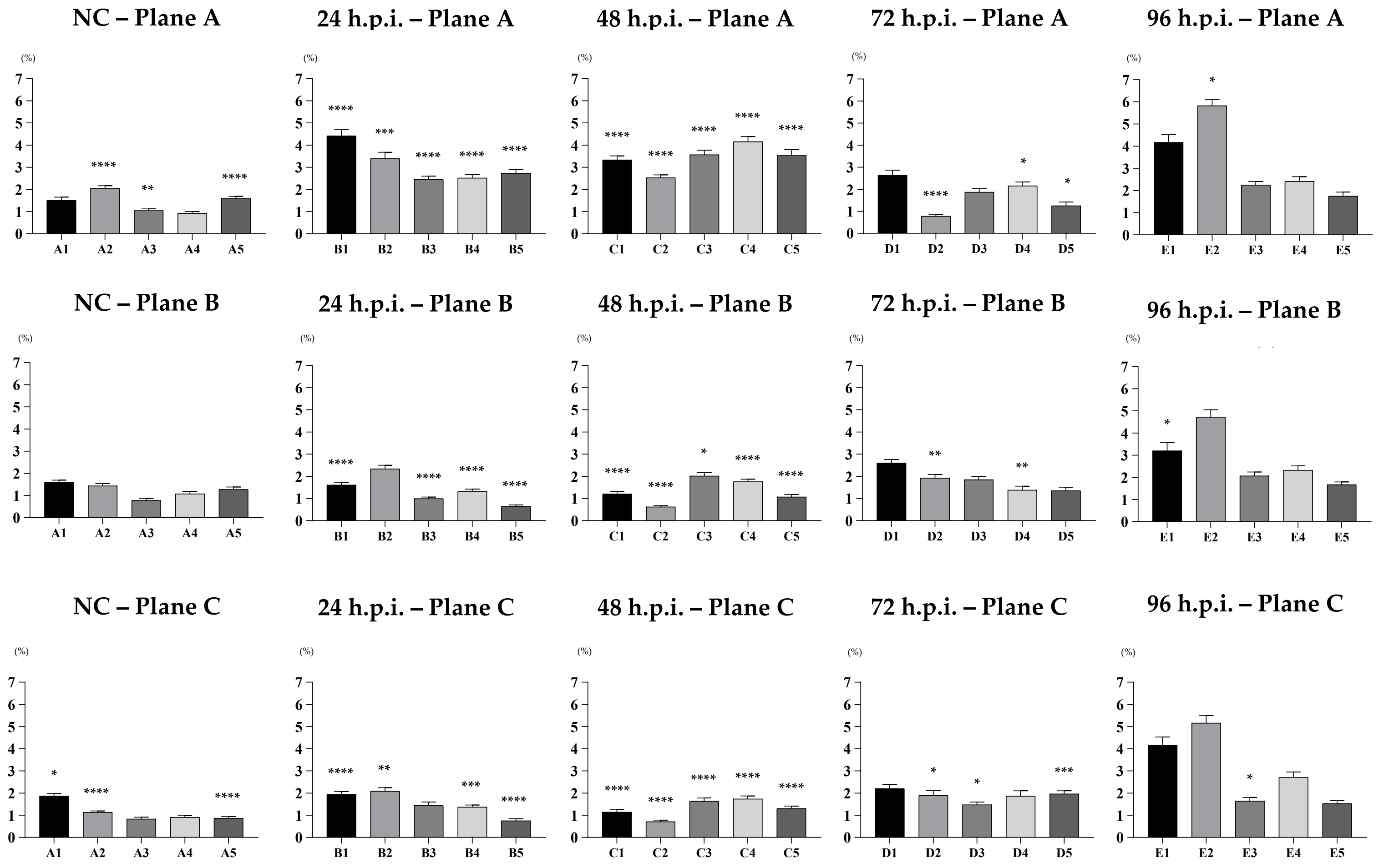


NC: Negative Control; h.p.i.: hours post-infection. Statistical analysis: unpaired t test with Welch's correction. *$p < 0.05$; **$p < 0.01$; ***$p < 0.001$; ****$p < 0.0001$.

# SINUSOIDAL DILATION – PART III

## Sinusoidal dilation – Thirty images from three sectioning planes

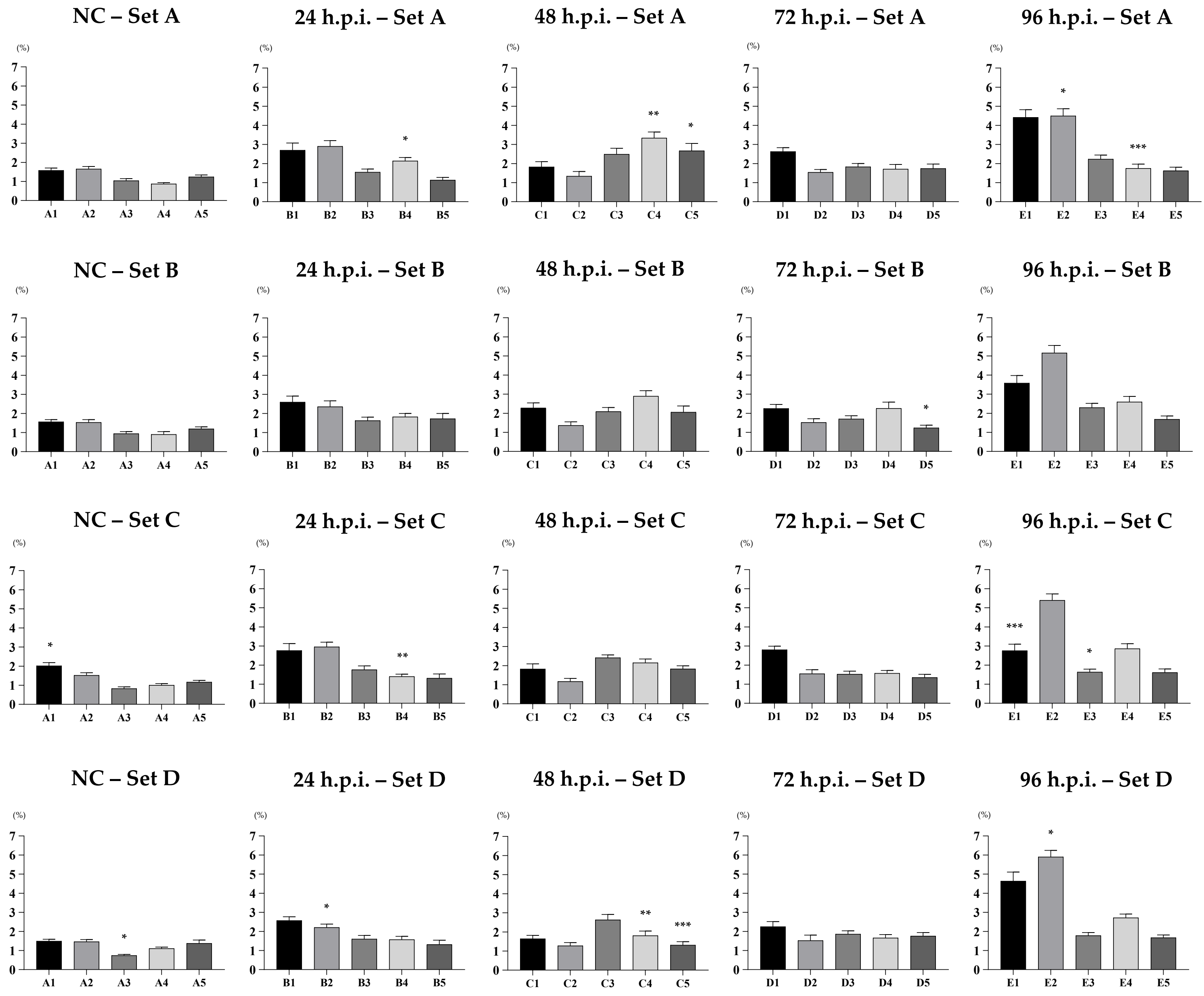


## Sinusoidal dilation – Total (120 images)

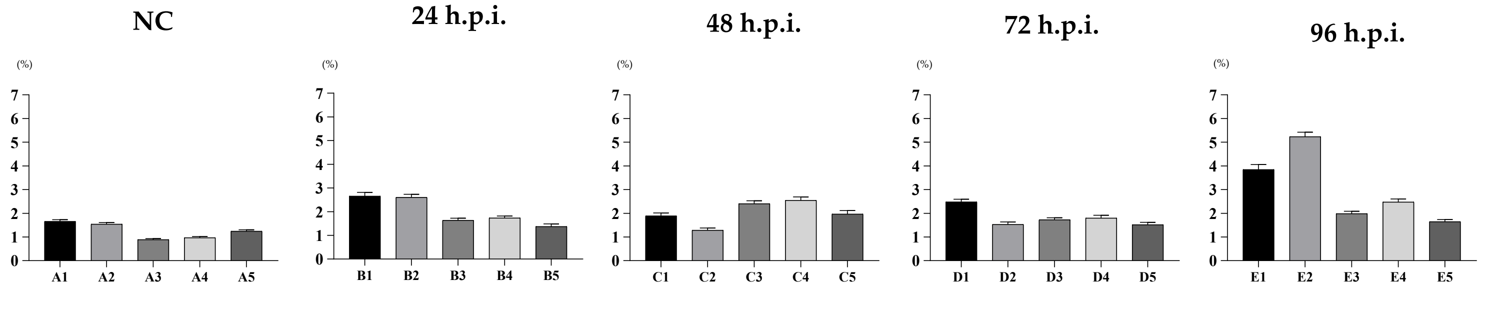


NC: Negative Control; h.p.i.: hours post-infection. Statistical analysis: unpaired t test with Welch's correction. *p < 0.05; **p < 0.01; ***p < 0.001; ****p < 0.0001.

# SINUSOIDAL DILATION – DESCRIPTIVE STATISTICS AND COMPARATIVE ANALYSIS NEGATIVE CONTROL – PART I

| | | | Animal 1 | Animal 2 | Animal 3 | Animal 4 | Animal 5 |
|---|---|---|---|---|---|---|---|
| Plane A | 10 Images | Subset Mean | 1.032 | 1.884 | 1.238 | 0.606 | 1.598 |
| | | (Total – Subset) Mean | 1.719 | 1.510 | 0.856 | 1.002 | 1.212 |
| | | p value | 0.0101 | > 0.05 | 0.0346 | 0.0004 | > 0.05 |
| | | Mean Difference ± SEM | 0.687 ± 0.221 | -0.374 ± 0.241 | -0.382 ± 0.158 | 0.396 ± 0.093 | -0.386 ± 0.196 |
| | 30 Images | Subset Mean | 1.509 | 2.084 | 1.094 | 0.8338 | 1.533 |
| | | (Total – Subset) Mean | 1.712 | 1.361 | 0.819 | 1.014 | 1.147 |
| | | p value | > 0.05 | < 0.0001 | 0.0100 | > 0.05 | 0.0034 |
| | | Mean Difference ± SEM | 0.203 ± 0.186 | -0.724 ± 0.154 | -0.275 ± 0.103 | 0.180 ± 0.110 | -0.386 ± 0.126 |
| | 40 Images | Subset Mean | 1.514 | 2.055 | 1.049 | 0.924 | 1.591 |
| | | (Total – Subset) Mean | 1.735 | 1.285 | 0.807 | 0.991 | 1.070 |
| | | p value | > 0.05 | < 0.0001 | 0.0094 | > 0.05 | < 0.0001 |
| | | Mean Difference ± SEM | 0.221 ± 0.155 | -0.770 ± 0.128 | -0.243 ± 0.091 | 0.067 ± 0.099 | -0.522 ± 0.117 |
| Plane B | 10 Images | Subset Mean | 1.843 | 1.782 | 0.897 | 1.064 | 1.381 |
| | | (Total – Subset) Mean | 1.645 | 1.520 | 0.887 | 0.961 | 1.231 |
| | | p value | > 0.05 | > 0.05 | > 0.05 | > 0.05 | 0.1736 |
| | | Mean Difference ± SEM | -0.198 ± 0.175 | -0.263 ± 0.280 | -0.010 ± 0.189 | -0.104 ± 0.161 | -0.150 ± 0.107 |
| | 30 Images | Subset Mean | 1.657 | 1.462 | 0.806 | 1.121 | 1.188 |
| | | (Total – Subset) Mean | 1.663 | 1.568 | 0.915 | 0.918 | 1.262 |
| | | p value | > 0.05 | > 0.05 | > 0.05 | > 0.05 | > 0.05 |
| | | Mean Difference ± SEM | 0.006 ± 0.131 | 0.106 ± 0.144 | 0.109 ± 0.115 | -0.202 ± 0.150 | 0.075 ± 0.102 |
| | 40 Images | Subset Mean | 1.603 | 1.439 | 0.781 | 1.075 | 1.274 |
| | | (Total – Subset) Mean | 1.690 | 1.593 | 0.941 | 0.915 | 1.229 |
| | | p value | > 0.05 | > 0.05 | > 0.05 | > 0.05 | > 0.05 |
| | | Mean Difference ± SEM | 0.087 ± 0.126 | 0.154 ± 0.133 | 0.160 ± 0.098 | -0.160 ± 0.122 | -0.045 ± 0.132 |

SEM: Standard Error of the Mean.

## SINUSOIDAL DILATION – DESCRIPTIVE STATISTICS AND COMPARATIVE ANALYSIS NEGATIVE CONTROL – PART II

| | | | Animal 1 | Animal 2 | Animal 3 | Animal 4 | Animal 5 |
|---|---|---|---|---|---|---|---|
| **Plane C** | **10 Images** | **Subset Mean** | 1.867 | 1.292 | 0.991 | 0.9418 | 0.737 |
| | | **(Total – Subset) Mean** | 1.643 | 1.564 | 0.878 | 0.9712 | 1.290 |
| | | **p value** | > 0.05 | > 0.05 | > 0.05 | > 0.05 | 0.0001 |
| | | **Mean Difference ± SEM** | -0.225 ± 0.176 | 0.273 ± 0.140 | -0.113 ± 0.208 | 0.029 ± 0.116 | 0.553 ± 0.115 |
| | **30 Images** | **Subset Mean** | 1.990 | 1.156 | 0.914 | 0.816 | 0.879 |
| | | **(Total – Subset) Mean** | 1.552 | 1.670 | 0.879 | 1.020 | 1.365 |
| | | **p value** | 0.0046 | < 0.0001 | > 0.05 | 0.0223 | < 0.0001 |
| | | **Mean Difference ± SEM** | -0.438 ± 0.148 | 0.514 ± 0.113 | -0.035 ± 0.110 | 0.203 ± 0.087 | 0.487 ± 0.103 |
| | **40 Images** | **Subset Mean** | 1.867 | 1.131 | 0.833 | 0.907 | 0.866 |
| | | **(Total – Subset) Mean** | 1.558 | 1.747 | 0.915 | 1.000 | 1.433 |
| | | **p value** | 0.0252 | < 0.0001 | > 0.05 | > 0.05 | < 0.0001 |
| | | **Mean Difference ± SEM** | -0.309 ± 0.135 | 0.616 ± 0.105 | 0.083 ± 0.096 | 0.093 ± 0.096 | 0.567 ± 0.102 |
| **30 Images Multi-plane Sets** | **Set A** | **Subset Mean** | 1.581 | 1.653 | 1.042 | 0.871 | 1.238 |
| | | **(Total – Subset) Mean** | 1.668 | 1.504 | 0.836 | 1.001 | 1.245 |
| | | **p value** | > 0.05 | > 0.05 | > 0.05 | > 0.05 | > 0.05 |
| | | **Mean Difference ± SEM** | 0.108 ± 0.146 | -0.148 ± 0.151 | -0.206 ± 0.115 | 0.131 ± 0.096 | 0.007 ± 0.123 |
| | **Set B** | **Subset Mean** | 1.559 | 1.530 | 0.945 | 0.903 | 1.195 |
| | | **(Total – Subset) Mean** | 1.696 | 1.545 | 0.869 | 0.991 | 1.260 |
| | | **p value** | > 0.05 | > 0.05 | > 0.05 | > 0.05 | > 0.05 |
| | | **Mean Difference ± SEM** | 0.137 ± 0.142 | 0.015 ± 0.161 | -0.076 ± 0.113 | 0.088 ± 0.153 | 0.065 ± 0.124 |
| | **Set C** | **Subset Mean** | 2.017 | 1.519 | 0.827 | 0.998 | 1.166 |
| | | **(Total – Subset) Mean** | 1.543 | 1.549 | 0.908 | 0.959 | 1.270 |
| | | **p value** | 0.011 | > 0.05 | > 0.05 | > 0.05 | > 0.05 |
| | | **Mean Difference ± SEM** | -0.474 ± 0.178 | 0.029 ± 0.156 | 0.080 ± 0.103 | -0.038 ± 0.107 | 0.103 ± 0.118 |
| | **Set D** | **Subset Mean** | 1.490 | 1.464 | 0.736 | 1.104 | 1.375 |
| | | **(Total – Subset) Mean** | 1.719 | 1.567 | 0.938 | 0.924 | 1.200 |
| | | **p value** | > 0.05 | > 0.05 | 0.0145 | > 0.05 | > 0.05 |
| | | **Mean Difference ± SEM** | 0.229 ± 0.129 | 0.104 ± 0.136 | 0.202 ± 0.081 | -0.181 ± 0.098 | -0.175 ± 0.181 |

SEM: Standard Error of the Mean.

# SINUSOIDAL DILATION – DESCRIPTIVE STATISTICS AND COMPARATIVE ANALYSIS 24 HOURS POST-INFECTION – PART I

| | | | Animal 1 | Animal 2 | Animal 3 | Animal 4 | Animal 5 |
|---|---|---|---|---|---|---|---|
| Plane A | 10 Images | Subset Mean | 5.064 | 3.905 | 2.394 | 3.128 | 2.037 |
| | | (Total – Subset) Mean | 2.437 | 2.485 | 1.565 | 1.608 | 1.312 |
| | | p value | 0.0016 | > 0.05 | 0.0235 | 0.0002 | 0.0003 |
| | | Mean Difference ± SEM | -2.626 ± 0.616 | -1.420 ± 0.661 | -0.829 ± 0.315 | -1.520 ± 0.273 | -0.725 ± 0.178 |
| | 30 Images | Subset Mean | 4.728 | 3.766 | 2.569 | 2.535 | 2.815 |
| | | (Total – Subset) Mean | 1.966 | 2.216 | 1.322 | 1.467 | 0.892 |
| | | p value | < 0.0001 | < 0.0001 | < 0.0001 | < 0.0001 | < 0.0001 |
| | | Mean Difference ± SEM | -2.763 ± 0.374 | -1.550 ± 0.358 | -1.247 ± 0.188 | -1.068 ± 0.202 | -1.923 ± 0.195 |
| | 40 Images | Subset Mean | 4.418 | 3.390 | 2.459 | 2.518 | 2.733 |
| | | (Total – Subset) Mean | 1.775 | 2.210 | 1.221 | 1.343 | 0.692 |
| | | p value | < 0.0001 | 0.0004 | < 0.0001 | < 0.0001 | < 0.0001 |
| | | Mean Difference ± SEM | -2.643 ± 0.302 | -1.181 ± 0.310 | -1.238 ± 0.164 | -1.175 ± 0.168 | -2.041 ± 0.175 |
| Plane B | 10 Images | Subset Mean | 1.407 | 2.487 | 0.987 | 1.824 | 0.877 |
| | | (Total – Subset) Mean | 2.770 | 2.614 | 1.693 | 1.726 | 1.418 |
| | | p value | < 0.0001 | > 0.05 | 0.0017 | > 0.05 | 0.0109 |
| | | Mean Difference ± SEM | 1.363 ± 0.282 | 0.127 ± 0.310 | 0.706 ± 0.188 | -0.098 ± 0.252 | 0.541 ± 0.195 |
| | 30 Images | Subset Mean | 1.530 | 2.347 | 1.017 | 1.483 | 0.718 |
| | | (Total – Subset) Mean | 3.023 | 2.679 | 1.844 | 1.822 | 1.595 |
| | | p value | < 0.0001 | > 0.05 | < 0.0001 | 0.0373 | < 0.0001 |
| | | Mean Difference ± SEM | 1.494 ± 0.229 | 0.333 ± 0.254 | 0.827 ± 0.135 | 0.339 ± 0.160 | 0.877 ± 0.167 |
| | 40 Images | Subset Mean | 1.605 | 2.336 | 0.997 | 1.318 | 0.636 |
| | | (Total – Subset) Mean | 3.182 | 2.737 | 1.952 | 1.943 | 1.741 |
| | | p value | < 0.0001 | > 0.05 | < 0.0001 | < 0.0001 | < 0.0001 |
| | | Mean Difference ± SEM | 1.576 ± 0.234 | 0.401 ± 0.244 | 0.955 ± 0.134 | 0.625 ± 0.149 | 1.105 ± 0.164 |

SEM: Standard Error of the Mean.

# SINUSOIDAL DILATION – DESCRIPTIVE STATISTICS AND COMPARATIVE ANALYSIS 24 HOURS POST-INFECTION – PART II

| | | | Animal 1 | Animal 2 | Animal 3 | Animal 4 | Animal 5 |
|---|---|---|---|---|---|---|---|
| Plane C | 10 Images | Subset Mean | 1.623 | 2.310 | 1.270 | 1.464 | 0.487 |
| | | (Total – Subset) Mean | 2.750 | 2.630 | 1.667 | 1.759 | 1.453 |
| | | p value | 0.0002 | > 0.05 | 0.0239 | > 0.05 | < 0.0001 |
| | | Mean Difference ± SEM | 1.127 ± 0.259 | 0.320 ± 0.455 | 0.397 ± 0.163 | 0.295 ± 0.186 | 0.966 ± 0.144 |
| | 30 Images | Subset Mean | 1.772 | 2.066 | 1.361 | 1.358 | 0.657 |
| | | (Total – Subset) Mean | 3.012 | 2.863 | 1.728 | 1.918 | 1.685 |
| | | p value | < 0.0001 | 0.0039 | 0.064 | 0.0004 | < 0.0001 |
| | | Mean Difference ± SEM | 1.240 ± 0.250 | 0.797 ± 0.268 | 0.368 ± 0.181 | 0.560 ± 0.153 | 1.028 ± 0.165 |
| | 40 Images | Subset Mean | 1.945 | 2.084 | 1.445 | 1.367 | 0.748 |
| | | (Total – Subset) Mean | 3.012 | 2.863 | 1.728 | 1.918 | 1.685 |
| | | p value | < 0.0001 | 0.0013 | > 0.05 | 0.0002 | < 0.0001 |
| | | Mean Difference ± SEM | 1.066 ± 0.252 | 0.779 ± 0.235 | 0.284 ± 0.188 | 0.550 ± 0.146 | 0.963 ± 0.175 |
| 30 Images Multi-plane Sets | Set A | Subset Mean | 2.698 | 2.900 | 1.550 | 2.139 | 1.134 |
| | | (Total – Subset) Mean | 2.642 | 2.504 | 1.662 | 1.600 | 1.452 |
| | | p value | > 0.05 | > 0.05 | > 0.05 | 0.0112 | > 0.05 |
| | | Mean Difference ± SEM | -0.056 ± 0.415 | -0.396 ± 0.331 | 0.112 ± 0.196 | -0.539 ± 0.204 | 0.318 ± 0.199 |
| | Set B | Subset Mean | 2.593 | 2.354 | 1.622 | 1.824 | 1.726 |
| | | (Total – Subset) Mean | 2.677 | 2.686 | 1.638 | 1.704 | 1.255 |
| | | p value | > 0.05 | > 0.05 | > 0.05 | > 0.05 | > 0.05 |
| | | Mean Difference ± SEM | 0.084 ± 0.366 | 0.332 ± 0.337 | 0.015 ± 0.213 | -0.120 ± 0.200 | -0.471 ± 0.296 |
| | Set C | Subset Mean | 2.764 | 2.953 | 1.760 | 1.401 | 1.318 |
| | | (Total – Subset) Mean | 2.620 | 2.487 | 1.592 | 1.846 | 1.391 |
| | | p value | > 0.05 | > 0.05 | > 0.05 | 0.0073 | > 0.05 |
| | | Mean Difference ± SEM | -0.143 ± 0.403 | -0.466 ± 0.291 | -0.169 ± 0.222 | 0.445 ± 0.161 | 0.073 ± 0.254 |
| | Set D | Subset Mean | 2.570 | 2.206 | 1.602 | 1.574 | 1.313 |
| | | (Total – Subset) Mean | 2.685 | 2.736 | 1.644 | 1.788 | 1.393 |
| | | p value | > 0.05 | 0.0301 | > 0.05 | > 0.05 | > 0.05 |
| | | Mean Difference ± SEM | 0.115 ± 0.281 | 0.530 ± 0.240 | 0.042 ± 0.213 | 0.214 ± 0.194 | 0.080 ± 0.264 |

SEM: Standard Error of the Mean.

# SINUSOIDAL DILATION – DESCRIPTIVE STATISTICS AND COMPARATIVE ANALYSIS 48 HOURS POST-INFECTION – PART I

| | | | Animal 1 | Animal 2 | Animal 3 | Animal 4 | Animal 5 |
|---|---|---|---|---|---|---|---|
| Plane A | 10 Images | Subset Mean | 3.574 | 2.955 | 4.493 | 5.197 | 5.253 |
| | | (Total – Subset) Mean | 1.739 | 1.134 | 2.215 | 2.307 | 1.668 |
| | | p value | 0.0012 | 0.0004 | < 0.0001 | 0.0003 | < 0.0001 |
| | | Mean Difference ± SEM | -1.835 ± 0.418 | -1.821 ± 0.355 | -2.278 ± 0.315 | -2.890 ± 0.543 | -3.584 ± 0.447 |
| | 30 Images | Subset Mean | 3.541 | 2.596 | 3.355 | 4.400 | 3.885 |
| | | (Total – Subset) Mean | 1.343 | 0.849 | 2.088 | 1.931 | 1.328 |
| | | p value | < 0.0001 | < 0.0001 | < 0.0001 | < 0.0001 | < 0.0001 |
| | | Mean Difference ± SEM | -2.198 ± 0.234 | -1.747 ± 0.171 | -1.266 ± 0.252 | -2.469 ± 0.292 | -2.558 ± 0.340 |
| | 40 Images | Subset Mean | 3.332 | 2.527 | 3.566 | 4.154 | 3.530 |
| | | (Total – Subset) Mean | 1.172 | 0.665 | 1.824 | 1.745 | 1.186 |
| | | p value | < 0.0001 | < 0.0001 | < 0.0001 | < 0.0001 | < 0.0001 |
| | | Mean Difference ± SEM | -2.159 ± 0.199 | -1.862 ± 0.139 | -1.742 ± 0.230 | -2.409 ± 0.243 | -2.345 ± 0.282 |
| Plane B | 10 Images | Subset Mean | 1.106 | 0.532 | 1.968 | 2.303 | 1.376 |
| | | (Total – Subset) Mean | 1.964 | 1.354 | 2.445 | 2.570 | 2.021 |
| | | p value | 0.0005 | < 0.0001 | > 0.05 | > 0.05 | 0.0384 |
| | | Mean Difference ± SEM | 0.858 ± 0.213 | 0.823 ± 0.145 | 0.477 ± 0.341 | 0.268 ± 0.254 | 0.645 ± 0.288 |
| | 30 Images | Subset Mean | 1.240 | 0.574 | 2.181 | 1.945 | 1.269 |
| | | (Total – Subset) Mean | 2.110 | 1.523 | 2.480 | 2.749 | 2.200 |
| | | p value | < 0.0001 | < 0.0001 | > 0.05 | 0.0003 | < 0.0001 |
| | | Mean Difference ± SEM | 0.870 ± 0.205 | 0.949 ± 0.134 | 0.299 ± 0.236 | 0.804 ± 0.217 | 0.931 ± 0.224 |
| | 40 Images | Subset Mean | 1.206 | 0.620 | 2.014 | 1.756 | 1.068 |
| | | (Total – Subset) Mean | 2.235 | 1.620 | 2.600 | 2.944 | 2.417 |
| | | p value | < 0.0001 | < 0.0001 | 0.0104 | < 0.0001 | < 0.0001 |
| | | Mean Difference ± SEM | 1.029 ± 0.198 | 0.999 ± 0.138 | 0.586 ± 0.225 | 1.188 ± 0.221 | 1.348 ± 0.224 |

SEM: Standard Error of the Mean.

## SINUSOIDAL DILATION – DESCRIPTIVE STATISTICS AND COMPARATIVE ANALYSIS 48 HOURS POST-INFECTION – PART II

| | | | Animal 1 | Animal 2 | Animal 3 | Animal 4 | Animal 5 |
|---|---|---|---|---|---|---|---|
| Plane C | 10 Images | Subset Mean | 0.793 | 0.532 | 1.007 | 2.524 | 1.381 |
| | | (Total – Subset) Mean | 1.992 | 1.354 | 2.532 | 2.550 | 2.020 |
| | | p value | < 0.0001 | < 0.0001 | < 0.0001 | > 0.05 | > 0.05 |
| | | Mean Difference ± SEM | 1.200 ± 0.176 | 0.823 ± 0.167 | 1.525 ± 0.209 | 0.027 ± 0.271 | 0.639 ± 0.306 |
| | 30 Images | Subset Mean | 1.144 | 0.698 | 1.454 | 2.042 | 1.404 |
| | | (Total – Subset) Mean | 2.142 | 1.482 | 2.722 | 2.717 | 2.155 |
| | | p value | < 0.0001 | < 0.0001 | < 0.0001 | 0.0037 | 0.0015 |
| | | Mean Difference ± SEM | 0.997 ± 0.219 | 0.783 ± 0.143 | 1.268 ± 0.212 | 0.675 ± 0.228 | 0.751 ± 0.231 |
| | 40 Images | Subset Mean | 1.139 | 0.711 | 1.635 | 1.734 | 1.303 |
| | | (Total – Subset) Mean | 2.269 | 1.573 | 2.790 | 2.955 | 2.299 |
| | | p value | < 0.0001 | < 0.0001 | < 0.0001 | < 0.0001 | < 0.0001 |
| | | Mean Difference ± SEM | 1.130 ± 0.204 | 0.863 ± 0.145 | 1.156 ± 0.208 | 1.221 ± 0.230 | 0.996 ± 0.231 |
| 30 Images Multi-plane Sets | Set A | Subset Mean | 1.824 | 1.339 | 2.490 | 3.341 | 2.670 |
| | | (Total – Subset) Mean | 1.915 | 1.268 | 2.377 | 2.284 | 1.733 |
| | | p value | > 0.05 | > 0.05 | > 0.05 | 0.0039 | 0.0275 |
| | | Mean Difference ± SEM | 0.091 ± 0.306 | -0.071 ± 0.265 | -0.113 ± 0.336 | -1.057 ± 0.346 | -0.937 ± 0.408 |
| | Set B | Subset Mean | 2.277 | 1.360 | 2.089 | 2.893 | 2.057 |
| | | (Total – Subset) Mean | 1.764 | 1.261 | 2.510 | 2.433 | 1.937 |
| | | p value | > 0.05 | > 0.05 | > 0.05 | > 0.05 | > 0.05 |
| | | Mean Difference ± SEM | -0.513 ± 0.295 | -0.099 ± 0.218 | 0.421 ± 0.256 | -0.460 ± 0.329 | -0.121 ± 0.365 |
| | Set C | Subset Mean | 1.824 | 1.169 | 2.411 | 2.152 | 1.831 |
| | | (Total – Subset) Mean | 1.915 | 1.325 | 2.403 | 2.680 | 2.012 |
| | | p value | > 0.05 | > 0.05 | > 0.05 | > 0.05 | > 0.05 |
| | | Mean Difference ± SEM | 0.092 ± 0.301 | 0.156 ± 0.203 | -0.007 ± 0.220 | 0.528 ± 0.261 | 0.182 ± 0.247 |
| | Set D | Subset Mean | 1.644 | 1.275 | 2.631 | 1.806 | 1.310 |
| | | (Total – Subset) Mean | 1.975 | 1.289 | 2.330 | 2.796 | 2.186 |
| | | p value | > 0.05 | > 0.05 | > 0.05 | 0.0012 | 0.0009 |
| | | Mean Difference ± SEM | 0.331 ± 0.235 | 0.014 ± 0.203 | -0.301 ± 0.311 | 0.990 ± 0.291 | 0.876 ± 0.255 |

SEM: Standard Error of the Mean.

# SINUSOIDAL DILATION – DESCRIPTIVE STATISTICS AND COMPARATIVE ANALYSIS 72 HOURS POST-INFECTION – PART I

| | | | Animal 1 | Animal 2 | Animal 3 | Animal 4 | Animal 5 |
|---|---|---|---|---|---|---|---|
| Plane A | 10 Images | Subset Mean | 1.741 | 1.000 | 1.867 | 2.261 | 2.184 |
| | | (Total – Subset) Mean | 2.550 | 1.580 | 1.718 | 1.758 | 1.462 |
| | | p value | > 0000.1 | 0.0097 | > 0.05 | > 0.05 | > 0.05 |
| | | Mean Difference ± SEM | 0.809 ± 0.171 | 0.580 ± 0.202 | -0.149 ± 0.371 | -0.503 ± 0.400 | -0.722 ± 0.537 |
| | 30 Images | Subset Mean | 2.287 | 0.895 | 1.937 | 2.333 | 1.267 |
| | | (Total – Subset) Mean | 2.548 | 1.744 | 1.661 | 1.622 | 1.607 |
| | | p value | > 0.05 | < 0.0001 | > 0.05 | 0.0055 | > 0.05 |
| | | Mean Difference ± SEM | 0.261 ± 0.266 | 0.848 ± 0.169 | -0.276 ± 0.198 | -0.711 ± 0.246 | 0.340 ± 0.240 |
| | 40 Images | Subset Mean | 2.646 | 0.777 | 1.871 | 2.156 | 1.249 |
| | | (Total – Subset) Mean | 2.401 | 1.909 | 1.660 | 1.622 | 1.659 |
| | | p value | > 0.05 | < 0.0001 | > 0.05 | 0.0210 | 0.0471 |
| | | Mean Difference ± SEM | -0.245 ± 0.256 | 1.133 ± 0.163 | -0.211 ± 0.183 | -0.535 ± 0.228 | 0.411 ± 0.203 |
| Plane B | 10 Images | Subset Mean | 3.137 | 2.110 | 1.750 | 1.743 | 1.156 |
| | | (Total – Subset) Mean | 2.423 | 1.479 | 1.728 | 1.805 | 1.556 |
| | | p value | 0.0495 | > 0.05 | > 0.05 | > 0.05 | > 0.05 |
| | | Mean Difference ± SEM | -0.714 ± 0.326 | -0.631 ± 0.328 | -0.022 ± 0.360 | 0.062 ± 0.522 | 0.400 ± 0.372 |
| | 30 Images | Subset Mean | 2.818 | 2.105 | 1.789 | 1.430 | 1.198 |
| | | (Total – Subset) Mean | 2.317 | 1.340 | 1.710 | 1.923 | 1.630 |
| | | p value | 0.0450 | 0.0010 | > 0.05 | > 0.05 | 0.0317 |
| | | Mean Difference ± SEM | -0.447 ± 0.219 | -0.765 ± 0.220 | -0.078 ± 0.194 | 0.493 ± 0.257 | 0.432 ± 0.196 |
| | 40 Images | Subset Mean | 2.600 | 1.925 | 1.848 | 1.381 | 1.352 |
| | | (Total – Subset) Mean | 2.424 | 1.335 | 1.671 | 2.009 | 1.607 |
| | | p value | > 0.05 | 0.0062 | > 0.05 | 0.0066 | > 0.05 |
| | | Mean Difference ± SEM | -0.176 ± 0.215 | -0.590 ± 0.210 | -0.177 ± 0.179 | 0.628 ± 0.226 | 0.255 ± 0.194 |

SEM: Standard Error of the Mean.

# SINUSOIDAL DILATION – DESCRIPTIVE STATISTICS AND COMPARATIVE ANALYSIS 72 HOURS POST-INFECTION – PART II

| | | | Animal 1 | Animal 2 | Animal 3 | Animal 4 | Animal 5 |
|---|---|---|---|---|---|---|---|
| Plane C | 10 Images | Subset Mean | 3.008 | 1.517 | 1.871 | 1.142 | 1.882 |
| | | (Total – Subset) Mean | 2.435 | 1.533 | 1.717 | 1.860 | 1.490 |
| | | p value | > 0.05 | > 0.05 | > 0.05 | 0.0259 | > 0.05 |
| | | Mean Difference ± SEM | -0.573 ± 0.411 | 0.016 ± 0.187 | -0.154 ± 0.227 | 0.718 ± 0.287 | -0.393 ± 0.271 |
| | 30 Images | Subset Mean | 2.575 | 1.607 | 1.335 | 1.778 | 1.864 |
| | | (Total – Subset) Mean | 2.452 | 1.507 | 1.862 | 1.807 | 1.408 |
| | | p value | > 0.05 | > 0.05 | 0.0020 | > 0.05 | 0.0223 |
| | | Mean Difference ± SEM | -0.123 ± 0.241 | -1.003 ± 0.229 | 0.526 ± 0.164 | 0.029 ± 0.320 | -0.456 ± 0.194 |
| | 40 Images | Subset Mean | 2.202 | 1.894 | 1.471 | 1.862 | 1.966 |
| | | (Total – Subset) Mean | 2.623 | 1.351 | 1.859 | 1.769 | 1.301 |
| | | p value | > 0.05 | 0.0331 | 0.0191 | > 0.05 | 0.0004 |
| | | Mean Difference ± SEM | 0.421 ± 0.232 | -0.543 ± 0.249 | 0.388 ± 0.163 | -0.093 ± 0.273 | -0.665 ± 0.180 |
| 30 Images Multi-plane Sets | Set A | Subset Mean | 2.629 | 1.543 | 1.830 | 1.715 | 1.741 |
| | | (Total – Subset) Mean | 2.434 | 1.528 | 1.697 | 1.828 | 1.450 |
| | | p value | > 0.05 | > 0.05 | > 0.05 | > 0.05 | > 0.05 |
| | | Mean Difference ± SEM | -0.195 ± 0.242 | -0.015 ± 0.200 | -0.133 ± 0.199 | 0.113 ± 0.272 | -0.291 ± 0.254 |
| | Set B | Subset Mean | 2.248 | 1.518 | 1.703 | 2.255 | 1.236 |
| | | (Total – Subset) Mean | 2.561 | 1.536 | 1.739 | 1.648 | 1.618 |
| | | p value | > 0.05 | > 0.05 | > 0.05 | > 0.05 | 0.0380 |
| | | Mean Difference ± SEM | 0.312 ± 0.254 | 0.018 ± 0.233 | 0.036 ± 0.191 | -0.607 ± 0.344 | 0.381 ± 0.180 |
| | Set C | Subset Mean | 2.803 | 1.547 | 1.528 | 1.571 | 1.353 |
| | | (Total – Subset) Mean | 2.376 | 1.527 | 1.797 | 1.876 | 1.579 |
| | | p value | > 0.05 | > 0.05 | > 0.05 | > 0.05 | > 0.05 |
| | | Mean Difference ± SEM | -0.427 ± 0.230 | -0.020 ± 0.248 | 0.269 ± 0.182 | 0.305 ± 0.207 | 0.226 ± 0.203 |
| | Set D | Subset Mean | 2.251 | 1.519 | 1.859 | 1.658 | 1.760 |
| | | (Total – Subset) Mean | 2.560 | 1.536 | 1.687 | 1.847 | 1.443 |
| | | p value | > 0.05 | > 0.05 | > 0.05 | > 0.05 | > 0.05 |
| | | Mean Difference ± SEM | 0.309 ± 0.290 | 0.017 ± 0.308 | -0.172 ± 0.199 | 0.189 ± 0.227 | -0.316 ± 0.211 |

SEM: Standard Error of the Mean.

**SINUSOIDAL DILATION – DESCRIPTIVE STATISTICS AND COMPARATIVE ANALYSIS 96 HOURS POST-INFECTION – PART I**

| | | | Animal 1 | Animal 2 | Animal 3 | Animal 4 | Animal 5 |
|---|---|---|---|---|---|---|---|
| Plane A | 10 Images | Subset Mean | 4.397 | 5.620 | 2.830 | 1.773 | 2.117 |
| | | (Total – Subset) Mean | 3.796 | 5.202 | 1.910 | 2.543 | 1.606 |
| | | p value | > 0.05 | > 0.05 | 0.0373 | > 0.05 | > 0.05 |
| | | Mean Difference ± SEM | -0.601 ± 0.600 | -0.418 ± 0.392 | -0.921 ± 0.385 | 0.770 ± 0.481 | -0.511 ± 0.479 |
| | 30 Images | Subset Mean | 3.835 | 5.560 | 2.385 | 2.180 | 1.953 |
| | | (Total – Subset) Mean | 3.850 | 5.129 | 1.854 | 2.579 | 1.547 |
| | | p value | > 0.05 | > 0.05 | 0.0173 | > 0.05 | > 0.05 |
| | | Mean Difference ± SEM | 0.015 ± 0.467 | -0.431 ± 0.384 | -0.531 ± 0.216 | 0.399 ± 0.283 | -0.406 ± 0.237 |
| | 40 Images | Subset Mean | 4.173 | 5.823 | 2.247 | 2.409 | 1.746 |
| | | (Total – Subset) Mean | 3.683 | 4.942 | 1.856 | 2.514 | 1.599 |
| | | p value | > 0.05 | 0.0189 | > 0.05 | > 0.05 | > 0.05 |
| | | Mean Difference ± SEM | -0.491 ± 0.444 | -0.884 ± 0.370 | -0.391 ± 0.200 | 0.104 ± 0.264 | -0.147 ± 0.203 |
| Plane B | 10 Images | Subset Mean | 4.465 | 4.128 | 2.173 | 2.035 | 1.497 |
| | | (Total – Subset) Mean | 3.790 | 5.337 | 1.969 | 2.519 | 1.662 |
| | | p value | > 0.05 | > 0.05 | > 0.05 | > 0.05 | > 0.05 |
| | | Mean Difference ± SEM | -0.675 ± 0.895 | 1.209 ± 0.704 | -0.203 ± 0.348 | 0.484 ± 0.377 | 0.165 ± 0.249 |
| | 30 Images | Subset Mean | 2.744 | 4.733 | 2.130 | 2.330 | 1.489 |
| | | (Total – Subset) Mean | 3.846 | 5.236 | 1.986 | 2.479 | 1.648 |
| | | p value | 0.0146 | > 0.05 | > 0.05 | > 0.05 | > 0.05 |
| | | Mean Difference ± SEM | 1.102 ± 0.436 | 0.504 ± 0.428 | -0.144 ± 0.227 | 0.149 ± 0.276 | 0.159 ± 0.156 |
| | 40 Images | Subset Mean | 3.200 | 4.723 | 2.070 | 2.324 | 1.671 |
| | | (Total – Subset) Mean | 4.169 | 5.493 | 1.944 | 2.557 | 1.637 |
| | | p value | 0.0326 | > 0.05 | > 0.05 | > 0.05 | > 0.05 |
| | | Mean Difference ± SEM | 0.969 ± 0.445 | 0.771 ± 0.394 | -0.126 ± 0.211 | 0.233 ± 0.254 | -0.034 ± 0.167 |

SEM: Standard Error of the Mean.

# SINUSOIDAL DILATION – DESCRIPTIVE STATISTICS AND COMPARATIVE ANALYSIS 96 HOURS POST-INFECTION – PART II

| | | | Animal 1 | Animal 2 | Animal 3 | Animal 4 | Animal 5 |
|---|---|---|---|---|---|---|---|
| Plane C | 10 Images | Subset Mean | 4.386 | 3.736 | 1.691 | 1.436 | 1.256 |
| | | (Total – Subset) Mean | 3.797 | 5.373 | 2.013 | 2.574 | 1.684 |
| | | p value | > 0.05 | > 0.05 | > 0.05 | 0.0060 | 0.0235 |
| | | Mean Difference ± SEM | -0.589 ± 0.768 | 1.636 ± 0.779 | 0.322 ± 0.371 | 1.138 ± 0.3435 | 0.427 ± 0.172 |
| | 30 Images | Subset Mean | 4.173 | 4.754 | 1.649 | 2.689 | 1.483 |
| | | (Total – Subset) Mean | 3.737 | 5.398 | 2.099 | 2.409 | 1.703 |
| | | p value | > 0.05 | > 0.05 | > 0.05 | > 0.05 | > 0.05 |
| | | Mean Difference ± SEM | -0.436 ± 0.458 | 0.644 ± 0.453 | 0.451 ± 0.233 | -0.279 ± 0.342 | 0.221 ± 0.196 |
| | 40 Images | Subset Mean | 4.165 | 5.161 | 1.642 | 2.704 | 1.527 |
| | | (Total – Subset) Mean | 3.687 | 5.274 | 2.159 | 2.366 | 1.709 |
| | | p value | > 0.05 | > 0.05 | 0.0115 | > 0.05 | > 0.05 |
| | | Mean Difference ± SEM | -0.478 ± 0.448 | 0.113 ± 0.400 | 0.517 ± 0.200 | -0.338 ± 0.285 | 0.181 ± 0.177 |
| 30 Images Multi-plane Sets | Set A | Subset Mean | 4.416 | 4.495 | 2.231 | 1.748 | 1.624 |
| | | (Total – Subset) Mean | 3.656 | 5.484 | 1.905 | 2.723 | 1.656 |
| | | p value | > 0.05 | 0.0257 | > 0.05 | 0.0004 | > 0.05 |
| | | Mean Difference ± SEM | -0.760 ± 0.476 | 0.989 ± 0.429 | -0.327 ± 0.241 | 0.975 ± 0.261 | 0.033 ± 0.211 |
| | Set B | Subset Mean | 3.577 | 5.161 | 2.294 | 2.591 | 1.685 |
| | | (Total – Subset) Mean | 3.936 | 5.262 | 1.884 | 2.442 | 1.636 |
| | | p value | > 0.05 | > 0.05 | > 0.05 | > 0.05 | > 0.05 |
| | | Mean Difference ± SEM | 0.359 ± 0.471 | 0.101 ± 0.443 | -0.410 ± 0.248 | -0.149 ± 0.322 | -0.049 ± 0.200 |
| | Set C | Subset Mean | 2.759 | 5.391 | 1.638 | 2.859 | 1.616 |
| | | (Total – Subset) Mean | 4.209 | 5.185 | 2.102 | 2.352 | 1.659 |
| | | p value | 0.0009 | > 0.05 | 0.0177 | > 0.05 | > 0.05 |
| | | Mean Difference ± SEM | 1.450 ± 0.418 | -0.206 ± 0.406 | 0.464 ± 0.191 | -0.506 ± 0.297 | 0.043 ± 0.208 |
| | Set D | Subset Mean | 4.633 | 5.899 | 1.782 | 2.718 | 1.669 |
| | | (Total – Subset) Mean | 3.584 | 5.015 | 2.054 | 2.399 | 1.641 |
| | | p value | > 0.05 | 0.0343 | > 0.05 | > 0.05 | > 0.05 |
| | | Mean Difference ± SEM | -1.049 ± 0.527 | -0.884 ± 0.407 | 0.273 ± 0.198 | -0.319 ± 0.244 | -0.027 ± 0.181 |

SEM: Standard Error of the Mean.

# DESCRIPTIVE STATISTICS OF TOTAL COUNTS (120 IMAGES)

| | | | Animal 1 | Animal 2 | Animal 3 | Animal 4 | Animal 5 |
|---|---|---|---|---|---|---|---|
| Hepatocyte Counting | Negative Control | Mean | 62.86 | 59.90 | 58.18 | 66.35 | 65.13 |
| | | SEM | 0.954 | 0.646 | 0.622 | 0.791 | 1.139 |
| | 24 h.p.i. | Mean | 76.31 | 75.73 | 85.33 | 81.57 | 81.23 |
| | | SEM | 1.012 | 0.800 | 0.801 | 1.052 | 0.834 |
| | 48 h.p.i. | Mean | 63.96 | 72.02 | 75.99 | 79.67 | 79.18 |
| | | SEM | 0.933 | 0.734 | 0.762 | 0.805 | 0.692 |
| | 72 h.p.i. | Mean | 45.82 | 42.65 | 43.56 | 43.39 | 43.90 |
| | | SEM | 0.626 | 0.795 | 0.818 | 0.625 | 0.534 |
| | 96 h.p.i. | Mean | 78.57 | 81.79 | 57.40 | 63.06 | 55.57 |
| | | SEM | 0.983 | 1.125 | 0.626 | 0.730 | 0.813 |
| Hepatocyte Binucleation | Negative Control | Mean | 24.65 | 25.22 | 26.13 | 25.24 | 27.51 |
| | | SEM | 0.740 | 0.726 | 0.554 | 0.788 | 0.751 |
| | 24 h.p.i. | Mean | 31.23 | 31.27 | 30.74 | 30.42 | 29.62 |
| | | SEM | 0.647 | 0.810 | 0.571 | 0.646 | 0.635 |
| | 48 h.p.i. | Mean | 33.77 | 33.24 | 34.59 | 34.85 | 31.47 |
| | | SEM | 0.649 | 0.684 | 0.627 | 0.550 | 0.708 |
| | 72 h.p.i. | Mean | 40.52 | 36.90 | 36.29 | 35.00 | 36.46 |
| | | SEM | 0.720 | 0.943 | 0.886 | 0.771 | 0.790 |
| | 96 h.p.i. | Mean | 25.98 | 27.22 | 28.55 | 25.03 | 26.92 |
| | | SEM | 0.598 | 0.687 | 0.618 | 0.590 | 0.724 |
| Sinusoidal Dilation | Negative Control | Mean | 1.661 | 1.541 | 0.888 | 0.969 | 1.244 |
| | | SEM | 0.066 | 0.065 | 0.046 | 0.050 | 0.060 |
| | 24 h.p.i. | Mean | 2.656 | 2.603 | 1.634 | 1.734 | 1.373 |
| | | SEM | 0.159 | 0.132 | 0.091 | 0.085 | 0.111 |
| | 48 h.p.i. | Mean | 1.892 | 1.286 | 2.405 | 2.548 | 1.967 |
| | | SEM | 0.124 | 0.096 | 0.123 | 0.141 | 0.145 |
| | 72 h.p.i. | Mean | 2.483 | 1.532 | 1.730 | 1.800 | 1.522 |
| | | SEM | 0.111 | 0.107 | 0.083 | 0.117 | 0.094 |
| | 96 h.p.i. | Mean | 3.846 | 5.236 | 1.986 | 2.479 | 1.648 |
| | | SEM | 0.212 | 0.185 | 0.097 | 0.126 | 0.086 |

SEM: Standard Error of the Mean.
